# Leaky Integrate-and-Fire Neuron Circuit Based on Floating-Gate Integrator

**DOI:** 10.3389/fnins.2016.00212

**Published:** 2016-05-23

**Authors:** Vladimir Kornijcuk, Hyungkwang Lim, Jun Yeong Seok, Guhyun Kim, Seong Keun Kim, Inho Kim, Byung Joon Choi, Doo Seok Jeong

**Affiliations:** ^1^Center for Electronic Materials, Korea Institute of Science and TechnologySeoul, South Korea; ^2^Department of Materials Science and Engineering, Seoul National University of Science and TechnologySeoul, South Korea; ^3^Department of Materials Science and Engineering, Seoul National UniversitySeoul, South Korea

**Keywords:** floating-gate integrator, leaky integrate-and-fire neuron, spiking neural network, synaptic transistor, spatial integration

## Abstract

The artificial spiking neural network (SNN) is promising and has been brought to the notice of the theoretical neuroscience and neuromorphic engineering research communities. In this light, we propose a new type of artificial spiking neuron based on leaky integrate-and-fire (LIF) behavior. A distinctive feature of the proposed FG-LIF neuron is the use of a floating-gate (FG) integrator rather than a capacitor-based one. The relaxation time of the charge on the FG relies mainly on the tunnel barrier profile, e.g., barrier height and thickness (rather than the area). This opens up the possibility of large-scale integration of neurons. The circuit simulation results offered biologically plausible spiking activity (<100 Hz) with a capacitor of merely 6 fF, which is hosted in an FG metal-oxide-semiconductor field-effect transistor. The FG-LIF neuron also has the advantage of low operation power (<30 pW/spike). Finally, the proposed circuit was subject to possible types of noise, e.g., thermal noise and burst noise. The simulation results indicated remarkable distributional features of interspike intervals that are fitted to Gamma distribution functions, similar to biological neurons in the neocortex.

## Introduction

Ongoing research efforts into spiking neural networks (SNNs) attempt to gain a better understanding of the brain (Gerstner and Kistler, 2002; Markram, 2006) and/or realize its “electronic replicas” that partially imitate brain functionalities such as learning and memory (Mead, 1990; Jeong et al., 2013; Merolla et al., 2014; Qiao et al., 2015). The former generally employs computational SNNs; a vast number of spiking neurons are simulated on computers in search of their behaviors relating to neuronal representation at both low and high levels (Markram, 2006). By contrast, the latter relates physically working hardware SNNs and their components, e.g., spiking neurons and synapses, in favor of real-time interaction with environments, which is referred to as neuromorphic engineering (Mead, 1990). When emulating an SNN with a vast number of neurons, the hardware SNN largely outperforms the computational SNN in terms of runtime, given the latter's need for substantial computational resources. The larger the SNN, the greater the severity of its need for computational resources. The hardware SNN is thus perhaps a good solution to this practical problem, if the artificial neurons and synapses capture their biological counterparts with high precision (Indiveri et al., 2011; Azghadi et al., 2014). The components with limited precision—only capturing the essence of their biological counterparts—can be engaged in neuromorphic systems that are endowed with several brain functionalities such as spatiotemporal recognition despite the component-wise disparity in detailed behavior (Eliasmith and Anderson, 2004; Eliasmith et al., 2012).

Essentially, neurons in a biological network communicate by spikes. The membrane potential of each neuron rises amid incident presynaptic spikes that cause excitatory postsynaptic currents (EPSCs) through the dendrites. That is, the membrane integrates the EPSCs until the membrane potential reaches a threshold for spiking. This procedure is referred to as integrate-and-fire (IF; Burkitt, 2006). This IF procedure lays the foundations of computational neuron models, e.g., the leaky IF neuron (LIF; Burkitt, 2006), Hodgkin-Huxley neuron (Hodgkin and Huxley, 1952), and Izhikevich neuron models (Izhikevich, 2003), and the corresponding hardware models (Mead, 1989; Indiveri et al., 2011; Lim et al., 2015). Among the models, the LIF neuron is one of the most widely used models in light of its simplicity.

In view of real-time interaction with physical environments, it is desirable that the hardware neuron spikes at a rate similar to that of the biological neuron (ca. < 100 Hz) given that each spike consumes a certain amount of power. The higher the activity in a given period of time, the more power the neuron consumes. Toward this end, the interspike interval (ISI) between neighboring spikes in time reaches a few tens of milliseconds, which requires a comparable R-C time constant within the framework of the LIF neuron. To put it precisely, a linear low-pass filter, i.e., integrator, in the LIF neuron needs to be endowed with a cutoff frequency below the minimum activity of the biological neuron. Signal integration can be realized in different integrators, e.g., Tau-cell (Edwards and Cauwenberghs, 2000), the subthreshold log-domain integrator by Arthur and Boahen (2004), and a differential pair integrator (Bartolozzi and Indiveri, 2007), and they are nicely reviewed in a paper written by Indiveri et al. (2011). For these integrators, a capacitor causes a delay in the response to an input signal so that the capacitance significantly alters the time delay, partly akin to an R-C delay in a simple R-C circuit.

The FG-based metal-oxide-semiconductor field-effect transistor (MOSFET), FG-MOSFET for short, is one of the most successfully commercialized nonvolatile memory bits in flash memory (Jeong et al., 2012). Remarkable progress in flash memory technology has been made, ranging from the charge trap flash as a variation of the FG-MOSFET to vertical NAND memory. The high maturity level of flash memory technology offers great opportunities for neuromorphic engineering; in particular, FG-MOSFETs are promisingly utilized as programmable synapses that work as local memories within a neuromorphic circuit (Hasler et al., 1994; Gordon et al., 2004; Tenore et al., 2006; Brink et al., 2013; Ramakrishnan et al., 2013). To date, diverse FG-MOSFET-based synapse circuits have been proposed with different precision; the simplest case is the single-transistor synapse device that can maintain the programmed synaptic weight for sufficiently long time periods and implement the spike-timing-dependent plasticity protocol (Hasler et al., 1994; Ramakrishnan et al., 2013). In addition, FG-MOSFETs are also employed as the core part of a synapse circuit (Tenore et al., 2006). Although synapse circuits containing FG-MOSFETs are diverse, it is common that the FG-MOSFETs are responsible for the memory of a programmed synaptic weight.

In this study, we propose an LIF neuron circuit based on a floating-gate (FG) integrator as a replacement for a capacitor integrator. Compared with FGs in synapse circuits, the role of an FG in this type of integrator is counterintuitive given that the FG is deliberately designed to retain the charge on the FG for a few seconds, at most. This poor charge retention is not acceptable in the FGs in synaptic circuits. Circuit simulations were conducted using LTspice IV in support of the proposed circuit. The kinetics of filling the floating gate with charge (charging) and emptying it (discharging) resembles the charging and discharging of a capacitor. However, a significant difference lies in the mechanism for charging and discharging. Charge transfer into and out of the FG is mainly determined by area-independent properties of the tunnel barrier, e.g., barrier height and thickness. Thus, the characteristic time constant—corresponding to that in a capacitor-based integrator—can be tweaked irrespective of the area of the FG, unlike the capacitor-based integrator. As a result, the circuit has excellent potential for scalability and very low power consumption.

## Materials and methods

### Circuit simulations

The circuit simulations were performed using LTspice IV. The LIF neuron circuit was designed by adopting 65-nm complementary metal-oxide-semiconductor (CMOS) technology that was implemented by using the BSIM 4.6.0 model (a built-in model in LTspice IV; Dunga et al., 2006). The parameters for all devices in this work can be found in Table 1. Quantum mechanical elastic tunneling through the tunnel barrier in a tunnel junction is a key phenomenon in the FG integrator; we utilized the tunneling equation included in the BSIM 4.6.0 model (Cao et al., 2000; Lee and Hu, 2001; Dunga et al., 2006). The tunneling equation is based on the Fowler-Nordheim tunneling within the framework of the Wentzel-Kramers-Brillouin approximation. The tunneling equation in the BSIM 4.6.0 model is semi-empirical with regard to the use of an auxiliary function that improves the accuracy of the original Fowler-Nordheim tunneling equation (Ranuárez et al., 2006).

**Table 1:** **Size of MOSFETs in use**.

	**Transistor number**	**Channel length (nm)**	**Channel width (nm)**	**Gate oxide thickness (nm)**
FG integrator	MT1–MT2	60	120	1.3
	M1	60	120	2.5
Amp1	M2–M5	60	240	2.5
Amp2	M6, M8	120	120	2.5
	M7, M9	60	120	2.5
Polarity inverter	M10	600	120	2.5
	M11	60	120	2.5
	M12, M13	120	120	2.5

### Noise implementation

Consecutive random number generation is required for simulating time-varying noise to be applied to the FGLIF neuron circuit. White voltage noise, e.g., thermal voltage noise, was simulated by generating an identical and independently distributed (i.i.d.) random number whose probability follows a normal distribution. The probability distribution function (PDF) is centered at zero with a standard deviation corresponding to the root-mean-square (RMS) amplitude of voltage noise (Δ*V*_*RMS*_). A new random number was repeatedly generated at each time bin (Δ*t*) from this PDF. The gate terminals of all MOSFETs in the neuron circuit were subject to such white noise.

Burst noise and flicker noise as a group of individual burst noises are nonwhite noise. Burst noise in an *n*-channel MOSFET, *n*MOS for short, is estimated to originate from repeated localization and delocalization of electrons by traps at the gate oxide/semiconductor interface (Hung et al., 1990). Both localization (trapping) and delocalization (detrapping) are stochastic and renewal processes with regard to the exponentially decaying PDF with the duration of an empty trap (τ_t_) and a filled trap (τ_det_) (Yonezawa et al., 2013). That is, the interaction between an electron and a trap is a Poisson process. First we defined the electron-trapping (detrapping) rate that evaluates the number of trapping (detrapping) events per unit time as *r*_*t*_ = 1/τ_t_ (*r*_det_ = 1/τ_det_). A uniform random number in the range between 0 and 1 was generated at each time step and compared with *r*_t_·Δ*t* to determine the occurrence of an electron-trapping event; the electron is trapped at the time step if the random number is smaller than *r*_t_·Δ*t*. The same holds for an electron-detrapping event except that the random number is compared with *r*_det_·Δ*t*. The time bin Δ*t* was sufficiently small (100 μs). For simplicity, it was assumed that *r*_t_ = *r*_det_.

The traps were assumed to be neutral if they were empty and located at the gate oxide/Si interface. Additionally, each trap was assumed to interact with only one electron. Each filled trap induces a change in the flat band voltage on average 〈Δ*V*_fb_〉 by $\langle \Delta V_{\text{fb}}\rangle =\alpha \cdot t_{\text{ox}}\cdot N_{a}^{0.6}∕\sqrt{L_{\text{eff}}\cdot W_{\text{eff}}}$, where α, *t*_ox_, *N*_a_, *L*_eff_, and *W*_eff_ denote an empirical constant, gate oxide thickness, acceptor density in the channel, and effective channel length and width, respectively (Fukuda et al., 2007). For all MOSFETs, α was set to 1.5 × 10^−12^ (Fukuda et al., 2007) and *N*_a_ to 1.7 × 10^17^ cm^−3^ (default value in the BSIM 4.6.0 model). Δ*V*_fb_ is generally distributed following an exponential PDF; the abovementioned 〈Δ*V*_fb_〉 denotes the expected, i.e., mean, value given the exponential PDF (Fukuda et al., 2007). In this regard, each trap was endowed with a particular random Δ*V*_fb_ value that was obtained from the exponential distribution. Note that Δ*V*_fb_ upon an electron-trapping event is positive given the appearance of a negative point charge at the gate oxide/Si interface.

Given the stochastic electron-trapping and detrapping processes, *V*_fb_ fluctuates with time following a Poisson process. The fluctuation in *V*_fb_ (Δ*V*_fb_) consequently alters the drain current (*I*_d_) at a given gate voltage (*V*_g_) with regard to the consequent change in the threshold voltage *V*_th_ (Δ*V*_th_), and Δ*V*_th_ = Δ*V*_fb_. The transconductance *g*_m_ is a function of the difference between *V*_g_ and *V*_th0_ + Δ*V*_th_, i.e., *g*_m_ = *f* {*V*_g_ − (*V*_th0_ + Δ*V*_th_)} = *f* {(*V*_g_ − Δ*V*_fb_) − *V*_th0_}, where *V*_th0_ denotes *V*_th_ in the absence of a trap. Imposing Δ*V*_fb_ on *V*_fb_ is thus equivalent to *V*_*g*_ subject to a fluctuation by −Δ*V*_fb_ with a noise-free *V*_th_, i.e., *V*_th0_. In our circuit simulations, the stochastic change in *V*_fb_ and the resulting change in *g*_m_ were simulated by changing *V*_g_ by −Δ*V*_fb_ at each time step. The aforementioned thermal noise was applied to *V*_g_ on top of this burst noise.

To simulate flicker noise, a group of *n* traps was assumed to simultaneously interact with electrons, and the interaction of each trap with an electron was independent of the others. The noise generation algorithm was analogous to the abovementioned burst noise generation except that the independent interactions of all *n* traps with electrons were simultaneously considered. The occurrence of trapping or detrapping at each trap at each time step was determined by comparing *r*_t_·Δ*t* or *r*_det_·Δ*t* with an i.i.d. random number. *r*_t_ for each trap was randomly chosen as for the abovementioned single-trap case. This comparison was repeated *n* times with *n* i.i.d. random numbers.

The same method was applied to *p*-channel MOSFETs, *p*MOS for short, regarding the interaction between a trap and a hole (rather than an electron). The donor density in the channel (*N*_d_) was 1.7 × 10^17^ cm^−3^, which is the default value in the BSIM 4.6.0 model. The main difference from the *n*MOS case lies in the charge of a filled trap, which is positive and negative for a *p*MOS and *n*MOS, respectively. Consequently, Δ*V*_fb_ for a *p*MOS upon a hole-trapping event is negative because of the appearance of a positive point charge at the gate oxide/Si interface, which is the opposite of the *n*MOS case. Thus, the equivalent change in *V*_g_ is also the opposite of the *n*MOS case.

## Results

### Circuit configuration

**Figure 1:**
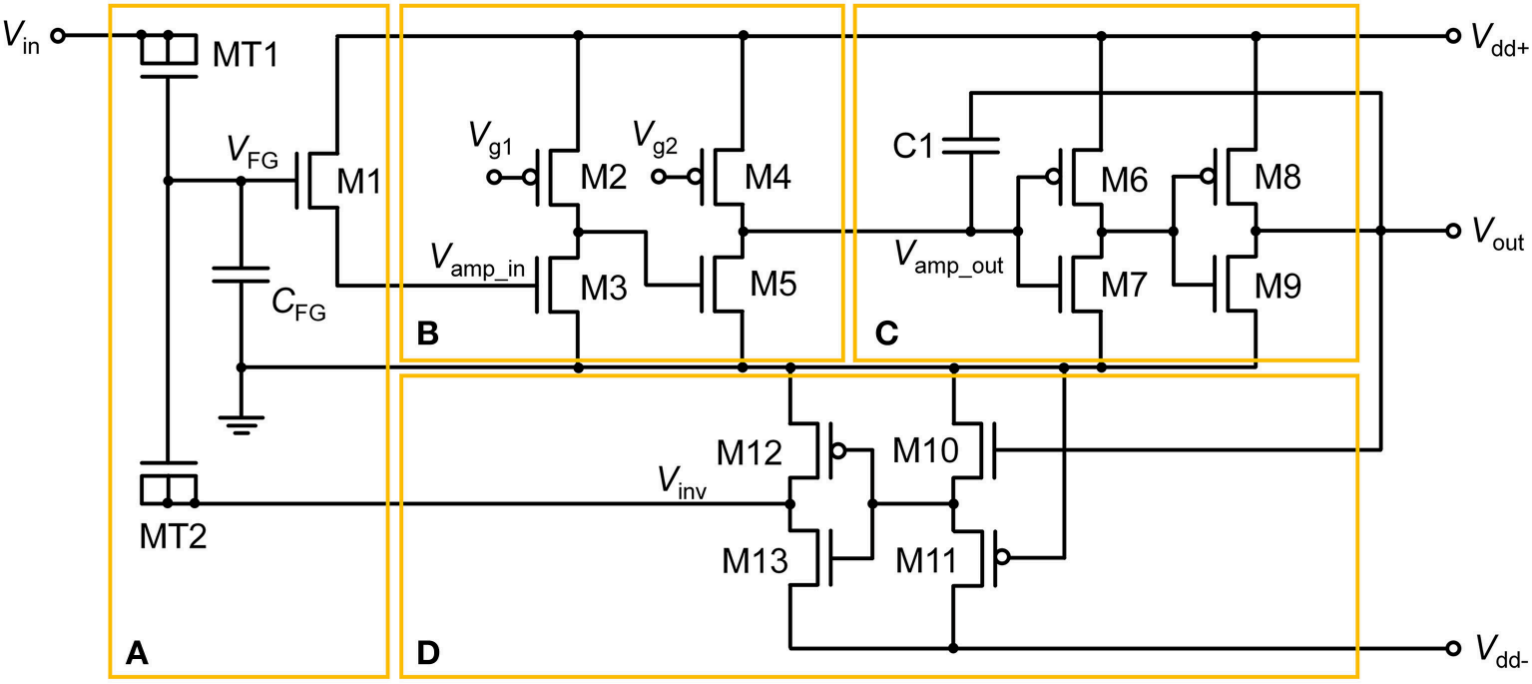
**FGLIF neuron circuit**. The circuit consists of four subcircuits: **(A)** FG integrator, **(B)** voltage amplifier, **(C)** voltage amplifier with positive feedback, and **(D)** polarity inverter.

The proposed floating-gate-based leaky integrate-and-fire (FGLIF) neuron circuit is depicted in Figure 1. The circuit consists of 12 MOSFETs (M2–M13), a single FG transistor (M1 + C_FG_), and a capacitor C1. The FG transistor has separate terminals MT1 and MT2 (tunnel junctions) for programming charge in the FG through quantum mechanical tunneling. Such an FG transistor is often referred to as a synapse transistor (Diorio et al., 1998; Rahimi et al., 2002). As shown in Figure 1, the circuit is divided into four subcircuits on functional grounds: (a) charge integrator, (b) non-inverting common source amplifier (Amp1), (c) non-inverting common-source amplifier (Amp2) with positive feedback, and (d) polarity inverter.

To begin, it is worth noting the synapse transistor (Figure 1A) that plays the key role in the proposed LIF neuron circuit. The additional terminals on MT1 and MT2 exclusively control the charge in the FG by means of quantum mechanical tunneling through the tunnel barriers in MT1 and MT2 given the use of a relatively thick gate oxide layer (2.5 nm) in FG transistor M1, which hinders the charge transfer through it. To put it precisely, the FG charge is controlled in a vertical tunnel junction of electrode-barrier-FG-barrier-electrode, following the prototypical synapse transistor proposed by Hasler et al. (1994). In the circuit simulator, the tunnel junctions are implemented by shorting the source, drain, and body terminals of a MOSFET, as shown in Figure 1A. Presynaptic currents are incident on the shorted terminal. All dielectrics in use in the circuit are SiO_2_ unless otherwise stated.

#### Integrator circuit

The integration in the circuit is realized by the FG integrator in Figure 1A. A charge is injected into the FG upon an incident presynaptic current on the tunnel junction and temporarily remains trapped. FG potential *V*_FG_ evolves in due course, which consequently changes the channel conductance of M1. *V*_FG_ thus parameterizes the integration. The injected charge simultaneously decays with time amid the presynaptic current injection, implying leaky integration that lays the foundation of LIF behavior in the FGLIF neuron. The retention time of an FG charge is mainly determined by the tunnel barrier thickness *d*_tun_ox_ and area, and the capacitance of C_FG_. The thick gate oxide of M1 barely intervenes in the charge ejection on the timescale of our interest. In contrast to an FG transistor as a nonvolatile memory element, our approach aims at the active use of charge-ejection dynamics; therefore, a charge retention time of approximately several seconds is desired. Accordingly, the circuit design and programming voltage are in need of tweaks. Several device parameters of the FG integrator in this work are listed in Table 1. The parameters in Table 2 were used for the circuit simulations unless otherwise stated.

**Table 2:** **Circuit parameters used for the circuit simulations**.

***V*_dd+_ (V)**	***V*_dd−_ (V)**	***V*_g1_ (V)**	***V*_g2_(V)**	**C_FG_ (fF)**	**C1 (fF)**
0.5	−0.5	0.7	0.65	6	0.15

**Figure 2:**
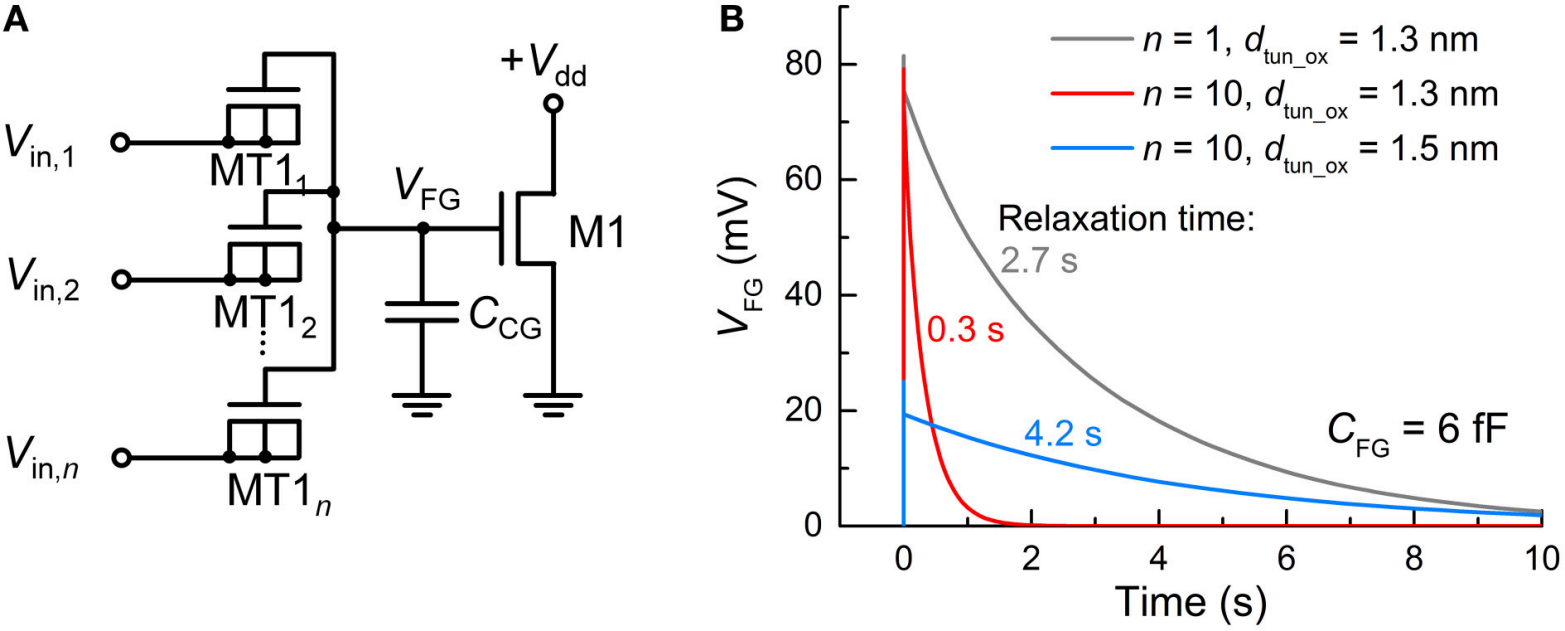
**FG integrator realizing spatial integration. (A)** FG integrator circuit. **(B)** Different relaxation behaviors of *V*_FG_—parameterizing integration—of the FG integrator for different *n* (1 and 10) and *d*_tun_ox_ values (1.3 and 1.5 nm). For all cases, the FG integrator was subject to an incident spike (*V*_in_ = 0.5 V, *t*_sp_ = 10 μs) on a single tunnel junction at 0 s while the others were grounded.

The synapse transistor has an advantage of employing multiple input tunnel junctions, enabling spatial integration (Polsky et al., 2004) that denotes the simultaneous integration of synaptic currents through different synapses. Figure 2A displays an FG integrator with *n* identical input terminals (MT1_1_–MT1_*n*_). For *n* = 10, a time-varying *V*_FG_ in response to an incident spike on a single terminal at 0 s was simulated with the other nine terminals being grounded. For comparison, the same simulation was conducted for *n* = 1. The input spike amplitude (*V*_in_) and width (*t*_sp_) were 0.5 V and 10 μs, respectively. Figure 2B relates the simulation results that uncover a decaying *V*_FG_ with different time constants, i.e., relaxation times, which depend on *n* and *d*_tun_ox_. The relaxation time is defined as the requisite time for *V*_FG_ to reach 1/*e* of *V*_FG_ at 0 s. The higher the number of input terminals, the shorter the relaxation time since each terminal works as a charge leakage path. Notably, a time constant of ca. 2.7 s for *n* = 1 is significantly reduced to 0.3 s for *n* = 10, as seen in Figure 2B. A workaround solution to such a reduction is to make use of thicker tunnel barriers, which offers a larger relaxation time (Figure 2B). The use of a thicker tunnel barrier trades off *V*_FG_ at 0 s for a larger relaxation time in light of the difficulty in charge injection upon spike arrival. Thus, one should carefully choose the tunnel barrier thickness that reconciles the charge relaxation (ejection) kinetics with the charge injection kinetics.

#### Amplifiers

MOSFETs M2–M5 in Figure 1B form a signal amplifier (Amp1) of two inverting common-source stages with *p*MOS loads. The input into Amp1 (*V*_amp_in_) is controlled by a voltage drop across the channel of M1, which is determined by *V*_FG_. That is, *V*_FG_ determines the output of Amp1. The output is subsequently relayed to the next amplifier (Amp2) in Figure 1C. The voltage-transfer characteristic (VTC) of each stage in Amp1 is controlled by constant gate voltages (*V*_g1_ and *V*_g2_); they are important in designing the output spike width and spiking threshold. We will set aside this issue until Section Adjustment of Circuit Parameters.

The signal exiting from Amp1 (*V*_amp_out_) enters Amp2, which directly elicits a spike at output terminal *V*_out_ when *V*_amp_out_ exceeds the transition region in the VTC of Amp2. The transition region is determined by two identical inverters (M6–M7 and M8–M9). The midpoint voltage, where *V*_out_ = *V*_amp_out_, is taken as a threshold voltage of Amp2 for spiking. Capacitor C1 realizes a positive capacitive feedback to the input to Amp2, allowing the output to remain high until the reset of the FG integrator. The reset occurs in rapid succession following the onset of spiking at *V*_out_ by negative feedback that is achieved by the polarity inverter in Figure 1D.

#### Polarity inverter

**Figure 3:**
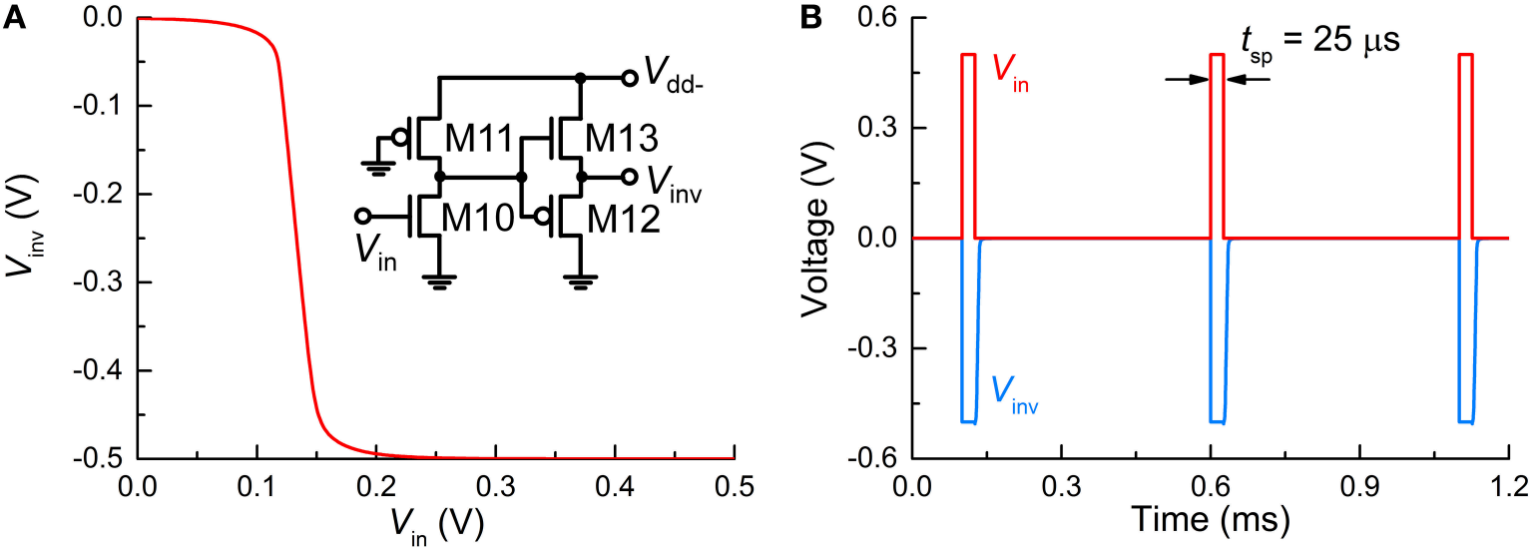
**Polarity inverter. (A)** VTC of the polarity inverter whose circuit is redrawn in the inset. **(B)** Output *V*_inv_ (blue solid line) in response to three input spikes (red solid line) in close succession (*V*_in_ = 0.5 V, *t*_sp_ = 25 μs).

The FG integrator is in need of a reset in order to complete a single spike. The reset is equivalent to emptying a charge in the FG by means of an electric field. The application of a negative voltage to terminal MT2 lets the previously injected charge vanish. Thus, a subcircuit that inverts *V*_out_ and relays it to the FG integrator is necessary. The subcircuit in Figure 1D, in conjunction with negative *V*_dd_ (*V*_dd−_), flips the polarity of *V*_out_, resulting in a negative output at *V*_inv_ in Figure 1D. To highlight this polarity inverter, the subcircuit is separately illustrated in Figure 3A, and the simulated VTC at a *V*_dd−_ of −0.5 V is plotted in Figure 3B. Note that *V*_in_ in Figure 3A corresponds to *V*_out_ in Figure 1. The VTC evidences a polarity reversal for *V*_in_ larger than the midpoint voltage. A negative *V*_inv_ pulse is accordingly elicited from the polarity inverter in response to a positive *V*_in_ pulse (0.5 V in amplitude and 25 μs in width), as seen in Figure 3B.

Provided that such a negative voltage pulse resets the FG integrator, *V*_FG_ consequently falls below zero. This reset process continues until *V*_FG_ becomes sufficiently low to let an input to either amplifier fall below the threshold for amplification.

### Circuit operation

#### Dc input mode

**Figure 4:**
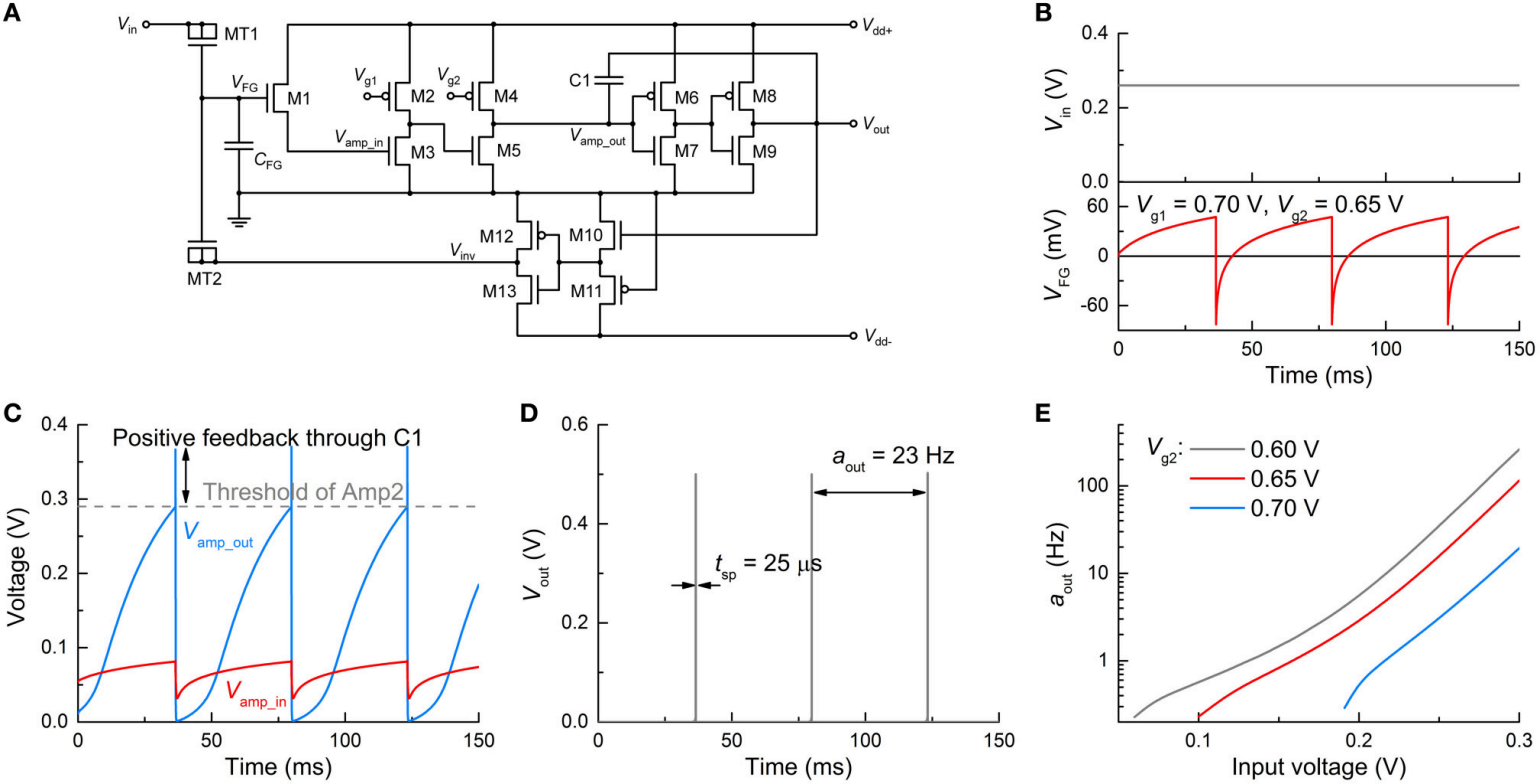
**Spiking characteristics in dc input mode. (A)** FGLIF neuron circuit (identical to Figure 1). **(B)** Input dc voltage *V*_in_ (upper panel) and resulting *V*_FG_ evolution (bottom panel). **(C)** Responses of *V*_amp_in_ and *V*_amp_out_ to *V*_in_, which correspond to the input and output of Amp1, respectively. The gray dashed line denotes the threshold of Amp2. The increase in *V*_amp_out_ owing to positive feedback through C1 is indicated. **(D)** Output spikes (*t*_sp_ = 25 μs, *a*_out_ = 23 Hz). **(E)** Neuronal gain function for three different *V*_g2_ values (0.6, 0.65, and 0.7 V).

First, we verify the spiking dynamics of the FGLIF neuron circuit (*n* = 1) under a constant voltage, which is equivalent to controlled neurophysiology experiments. Applying a constant voltage to MT1 continuously elevates *V*_FG_ (integration) in light of the positive charge injection into the FG. With that said, the detailed balance (Riggert et al., 2014) eventually reconciles the charge injection with the ejection at a particular *V*_FG_ level; therefore, the rate of a *V*_FG_ increase largely declines when it is close to this level. By contrast, an R-C integrator maintains such a balance through charging on C and simultaneous discharging through R. Figure 4B shows the *V*_FG_ variation in time at 0.26 V amid output spiking. *V*_FG_ rises in the first place, and thus so does the channel conductance of M1. *V*_amp_in_ and the resulting *V*_amp_out_ consequently increase until *V*_amp_out_ reaches the threshold (ca. 0.29 V) for high *V*_out_ through Amp2, as plotted in Figure 4C. The high *V*_out_ is temporally maintained in view of the positive feedback through C1. The high *V*_out_ simultaneously triggers the polarity inverter that activates negative feedback to the FG integrator, resetting the FG integrator. *V*_amp_out_ therefore falls below the threshold, leading to the termination of the high *V*_out_ and negative feedback. This procedure produces a single spike and is repeated for the next spike within the ISI. The reset rate determines the spike width: the faster the reset, the narrower the spike width. This relationship will be addressed in detail in Section Adjustment of Circuit Parameters. The simulation with the parameters in Table 1 uncovered an output spike width of 25 μs and an output activity *a*_out_ (spiking frequency) of ~23 Hz (Figure 4D).

The neuronal gain function is the substrate of encoding neuronal information (Gerstner and Kistler, 2002; Eliasmith and Anderson, 2004); different inputs are encoded to represent distinguishable outputs. In an attempt to verify a gain function of the FGLIF neuron, the neuronal activity was evaluated at different constant voltages (Figure 4E). A higher input voltage significantly speeds up FG charging within the ISI, reducing the ISI to a large extent. Thus, the activity largely increases with the input voltage. Figure 4E also uncovers a threshold input for spiking (60, 100, and 190 mV for *V*_g2_ = 0.60, 0.65, and 0.70 V, respectively), which is determined by the threshold of Amp2. Furthermore, there exists a minimum activity of ~0.2 Hz, and it features the type-II excitability of the Hodgkin-Huxley model (Dayan and Abbott, 2001). The minimum activity is fairly negligible. Note that the exponential change in activity upon voltage (Figure 4E) arises from the tunnel current that exponentially varies upon voltage (Jeong and Hwang, 2005; Soni et al., 2014).

#### Spiking input mode

**Figure 5:**
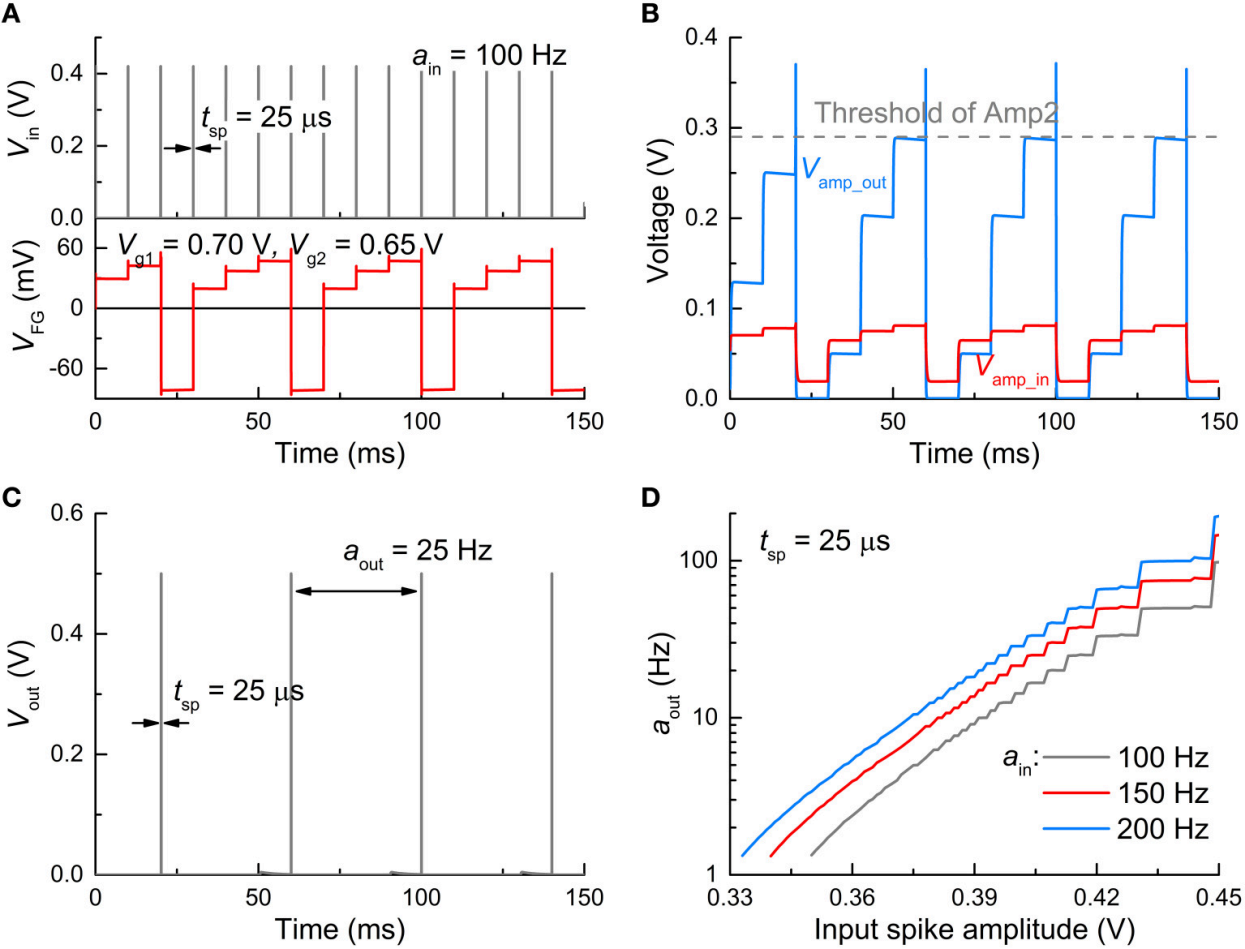
**Spiking characteristics in spiking input mode. (A)** Input signal profile (*V*_*in*_ = 0.42 V, *t*_sp_ = 25 μs) (upper panel) and resulting *V*_FG_ evolution (bottom panel). The input activity (*a*_in_) was set to 100 Hz. **(B)** Responses of *V*_amp_in_ and *V*_amp_out_ to the input spikes. **(C)** Output spikes (*t*_sp_= 25 μs, *a*_out_ = 25 Hz). **(D)** Neuronal gain function for three *a*_in_ values (100, 150, and 200 Hz).

A spiking input mode realizes practical circumstances for the operation of an FGLIF neuron in an SNN. As a whole, the response of the FGLIF neuron is comparable to the dc input mode, although there are differences to some extent. The subcircuit-wise responses to an input spike train (activity: 100 Hz; spike amplitude: 0.42 V; spike width: 25 μs) are plotted in Figure 5 in the same order as Figure 4. The main difference between the two modes lies in the integration: the input spikes cause stepwise evolution of *V*_FG_, unlike the former case (Figure 5A). However, irrespective of an input type, the FG integrator successfully features leaky integration. Analogous to the dc input mode, *V*_amp_in_ is amplified through Amp1 (Figure 5B), and a spike is elicited from *V*_out_ when *V*_amp_out_ crosses the threshold of Amp2 (Figure 5C). Likewise, the output activity differs for different spike amplitudes and activities, as shown in Figure 5D; the larger the amplitude or/and activity, the more frequently the neuron spikes. That is, when information transmission between pre- and postsynaptic neurons is invoked in an SNN, the postsynaptic FGLIF neuron is able to represent the presynaptic neuron's activity, i.e., input activity for the postsynaptic neuron, by outputting a distinguishable activity.

### Adjustment of circuit parameters

The proposed circuit provides a means of tweaking neuronal behavior such as relaxation time, spiking threshold, and spike width and amplitude. Recalling the change of the relaxation time upon the tunnel barrier thickness (Section Circuit Simulations), a thicker tunnel barrier, e.g., 1.5 nm for *n* = 10, is *a priori* preferred in favor of a relaxation timescale that is biologically plausible. The consequent decrease in maximum *V*_FG_ can be compensated for by increasing the spike width. For instance, for *d*_tun_ox_ = 1.5 nm, the use of a wider spike (100 μs) raises the maximum *V*_FG_ by approximately one order of magnitude, per our simulation (not shown).

**Figure 6:**
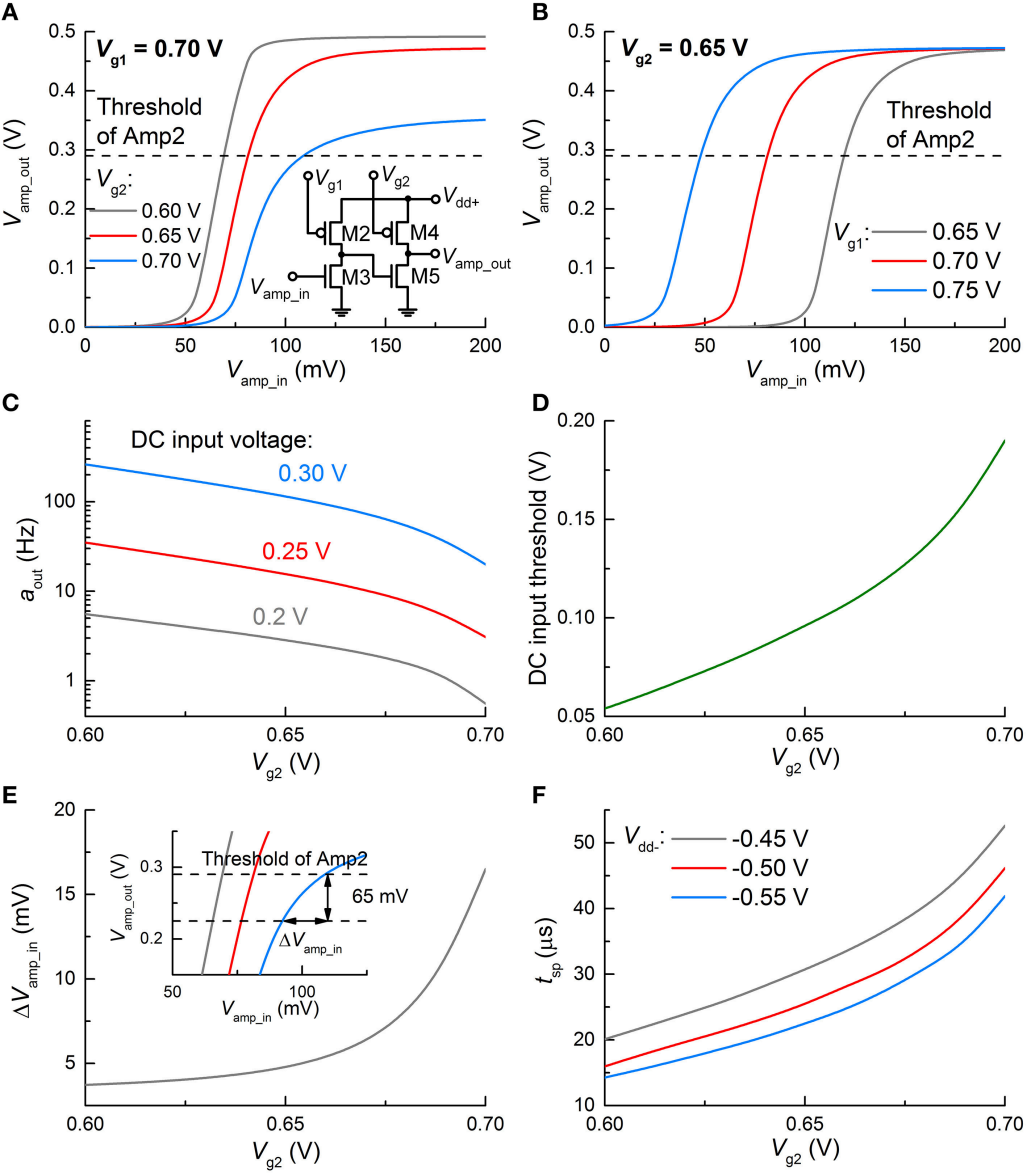
**Change in spiking dynamics upon circuit parameters**. Change in VTC of Amp1 **(A)** for three *V*_g2_ values and the same *V*_g1_ (0.7 V), and **(B)** for three *V*_g1_ and the same *V*_g2_ (0.65 V). **(C)** Change in *a*_out_ upon *V*_g2_ at 0.7 V *V*_g1_ for different input dc voltages (0.2, 0.25, and 0.3 V). **(D)** Threshold of *V*_in_ for spiking with *V*_g2_. **(E)** Requisite change in *V*_amp_in_ for ceasing spiking (Δ*V*_amp_in_) with *V*_g2_. The inset provides a graphical view of Δ*V*_amp_in_ for the VTCs **(A)** in case of an overshoot of 65 mV by the positive feedback. **(F)** Output spike width with *V*_g2_ for different *V*_dd−_ values (−0.45, −0.5, and −0.55 V).

In addition, gate voltages *V*_g1_ and *V*_g2_ of the loads in Amp1 alter the VTC. For instance, Figure 6A shows the VTC of Amp1 for three different *V*_g2_ values at the same *V*_g1_ (0.7 V) where significant changes in the VTC are seen. The same holds for *V*_g1_ as shown in Figure 6B; however, the VTC merely shifts relying on *V*_g1_. Provided that high *V*_out_ (spiking) is triggered only if *V*_amp_out_ reaches the threshold of Amp2 (denoted by a dashed line in Figure 6A), *V*_amp_in_ for spiking substantially varies upon *V*_g2_. In Figure 6A, a higher *V*_g2_ leads to a higher *V*_amp_in_ for spiking; therefore, spiking requires a higher *V*_FG_. A higher *V*_FG_ is in need of a longer integration time at a given input voltage in the dc input mode, or equivalently at a given input activity (if sufficiently high to evoke a spike) in the spiking input mode. Thus, output activity *a*_out_ declines with *V*_g2_ (Figure 6C). On the same grounds, a higher *V*_FG_ requires a higher *V*_in_ to output the same activity. As a consequence, the threshold for spiking in the dc input mode increases with *V*_g2_, as shown in Figure 6D. Therefore, the neuronal gain function can be easily modified by tweaking *V*_g2_.

Triggering the positive feedback through C1 elevates *V*_amp_out_ over the threshold of Amp2, as seen in Figures 4C, 5B; the overshoot (ca. 65 mV in this work) is mainly determined by the capacitance of C1 and *V*_dd+_. The overshoot is independent of *V*_g2_. Then *V*_amp_in_ immediately declines upon the onset of the negative feedback to the FG integrator through the polarity inverter. *V*_amp_in_ eventually falls below the threshold of Amp2. The requisite time for this process is equivalent to the spike width. The *V*_amp_out_-*V*_amp_in_ relations in Figure 6A indicate that the higher *V*_g2_ is given, the larger decrease in *V*_amp_in_ (Δ*V*_amp_in_) needs to be made by the negative feedback to drag *V*_amp_out_ below the threshold. Δ*V*_amp_in_ is elucidated in the inset of Figure 6E, where the VTCs in Figure 6A are zoomed in. Notably, the requisite Δ*V*_amp_in_ increases with *V*_g2_, and thus it takes longer for a higher *V*_g2_ to decrease *V*_amp_out_ by 65 mV, i.e., to reset the FG integrator with the same negative *V*_inv_. As a consequence, a higher *V*_g2_ offers a wider spike, as shown in Figure 6F. In addition, given that a higher |*V*_dd−_|evokes a higher |*V*_inv_|, resetting the FG integrator takes less time with a higher |*V*_dd−_|, rendering the output spike width narrower (Figure 6F).

### Power consumption

**Figure 7:**
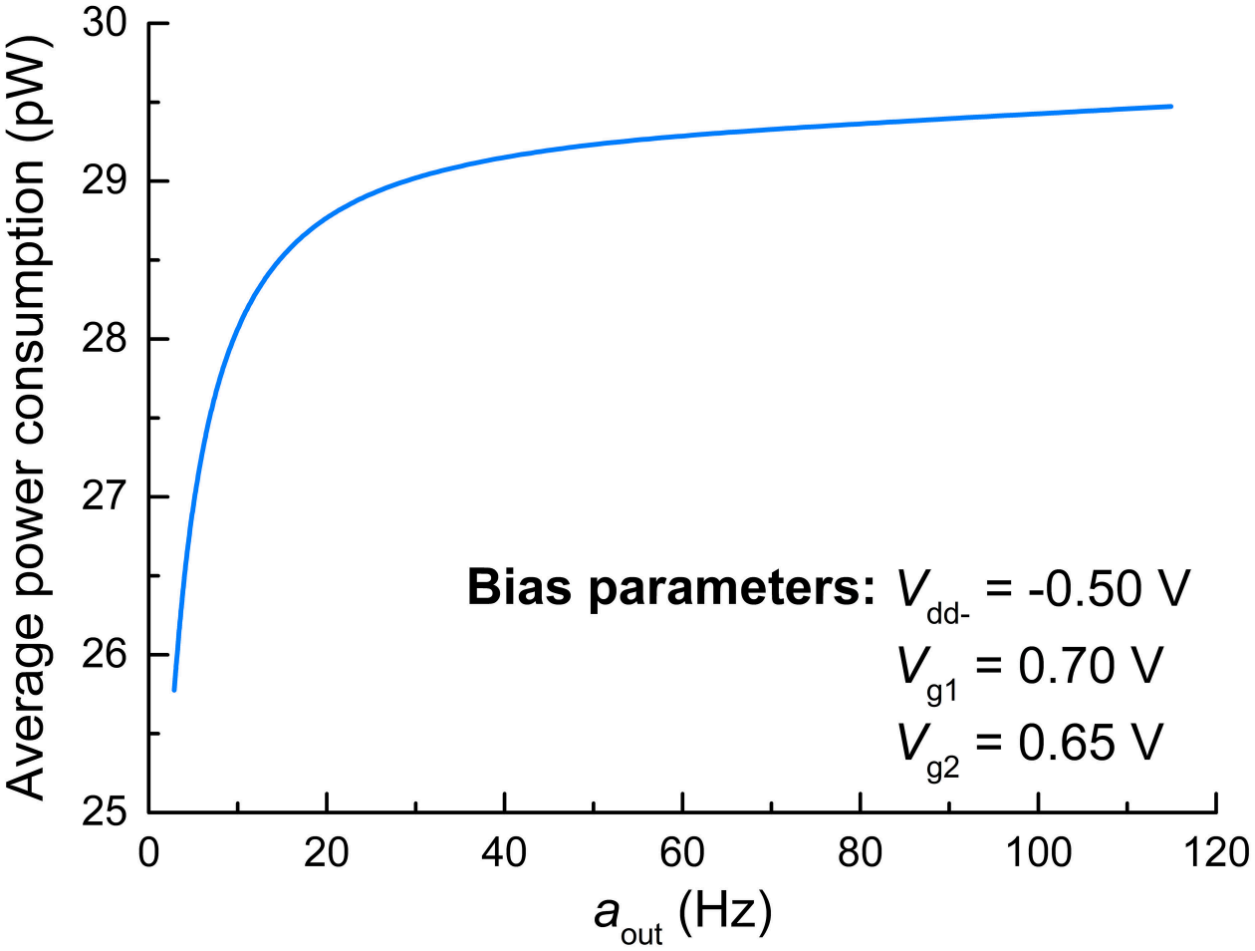
**Average power consumption of FGLIF neuron with respect to *a*_out_**.

Regarding the principles of neuromorphic engineering, low power consumption is strongly desired. Toward this end, we evaluated the power consumption of the FGLIF neuron that elicits different output activities amid the application of a constant input voltage. The average power consumption was acquired for various output activities by evaluating the consumed energy during the period of a single spike and dividing it by the period. The results are plotted in Figure 7, in which the average power consumption is gently proportional to the output activity. Notably, the proposed FGLIF neuron circuit consumes power less than 30 pW in the entire activity range. This is mainly ascribed to the subthreshold operation of Amp1 and Amp2, allowing a low current flow through the channels in series.

Power consumption of the proposed circuit was compared to the values achievable in the alternative VLSI neuron designs, which are listed in Table 3. The FGLIF neuron circuit provides power consumption that is several orders of magnitude lower than those of other models, while the number of transistors in use is comparable. Of course, a precise comparison between the circuits is still difficult since power consumption also strongly depends on several other factors such as CMOS technology in use, neuron spike width, and firing rate. Therefore, the results in Table 3 provide only an approximate overview and can be expected to change.

**Table 3:** **Power consumption comparison between the proposed neuron circuit and other VLSI neuron models**.

**Neuron model**	**Number of transistors used**	**Power consumption**	**References**
Conductance-based	27–30+	60 μW	Mahowald and Douglas, 1991
Integrate-and-Fire	18–20	>10 μW for output firing rate of 100 Hz	Indiveri et al., 2006
Hindmarsh-Rose	90	163.4 μW	Lee et al., 2004
Quadratic Integrate-and-Fire	14	8–40 μW	Wijekoon and Dudek, 2008
Log-domain low pass filter neuron	16	50–1000 nW	Arthur and Boahen, 2007
Log-domain Izhikevich neuron	17+	2.6 μW at rest state	Van Schaik et al., 2010
FGLIF	13	<30 pW	

### Spiking in the presence of noise

The operation of the FGLIF neuron circuit appears to be markedly susceptible to noise because of the subthreshold operation of the MOSFETs in the circuit. Thus, it is important to identify the effect of noise on the operation of the neuron circuit and analyze it in comparison with its biological counterpart. Types of noise in a MOSFET are (i) thermal noise, (ii) shot noise, (iii) burst noise, and (iv) flicker noise. The first two are white noise, whereas the last two are nonwhite noise whose power spectral density (PSD) relies on frequency (Chong and Sansen, 2013; Hamilton et al., 2014). The thermal voltage noise across a MOSFET channel is analogous to that across a resistor, and its PSD is *S*_*v*_ = 4*kTR*_d_ (Nyquist, 1928). Here *R*_d_ denotes the resistance of the channel. In the FGLIF circuit, the thermal noise in each CMOS stage (a pair of *p*MOS and *n*MOS channels) is filtered by the capacitance of the following stage. Therefore, the thermal noise endows each voltage input node with Δ*V*_RMS_:

(1) $$\begin{array}{l} \Delta V_{RMS}=\sqrt{k_{b}T∕C}, \end{array}$$

where *k*_*b*_, *T*, and *C* denote the Boltzmann constant, temperature, and equivalent MOS capacitance of the following stage, respectively. Note that the MOS capacitance relies on the gate oxide capacitance and gate voltage. Given the subthreshold operation of the MOSFETs in the neuron circuit, *C* is smaller than that of above-threshold-working MOSFETs, and thus the thermal noise effect is perhaps prominent with regard to Equation (1). For simplicity, *C* was evaluated at the average *V*_g_ values that are applied to the input of each CMOS stage during the circuit's operation. Δ*V*_*RMS*_ was evaluated at each node that precedes a given capacitance value, and it varied from 0.8 to 5.4 mV for different nodes. This thermal noise was taken into account in the following circuit simulations, as detailed in Section Materials and Methods. Additionally, it should be mentioned that Equation (1) is in fact not limited to thermal noise but is rather universal for the entire white noise of the system in the presence of a filtering capacitor (Sarpeshkar et al., 1993). As a result, no extra terms are needed to evaluate the shot noise in the circuit.

Burst noise (also known as random telegraph noise) appears to markedly affect the operation of the neuron circuit, given the fluctuation of *V*_fb_, i.e., Δ*V*_fb_, upon the interaction of a charge trap with an electron. The contribution of each trap interacting with an electron to 〈Δ*V*_fb_〉 differs for the MOSFETs in the circuit owing to the different channel areas and gate oxide thicknesses (see Section Materials and Methods). In the following simulations, 〈Δ*V*_fb_〉 for each MOSFET was evaluated, and the fluctuation of *V*_g_ (equivalent to Δ*V*_fb_ that was randomly generated from an exponential distribution function with 〈Δ*V*_fb_〉) was applied to each MOSFET following the Poisson process that is elucidated in Section Materials and Methods. The same held for M1 (FG-MOSFET) except that Δ*V*_fb_ was imposed on *V*_FG_ rather than *V*_g_. The electron-trapping rate *r*_t_ was also an i.i.d. random variable (see Section Materials and Methods).

**Figure 8:**
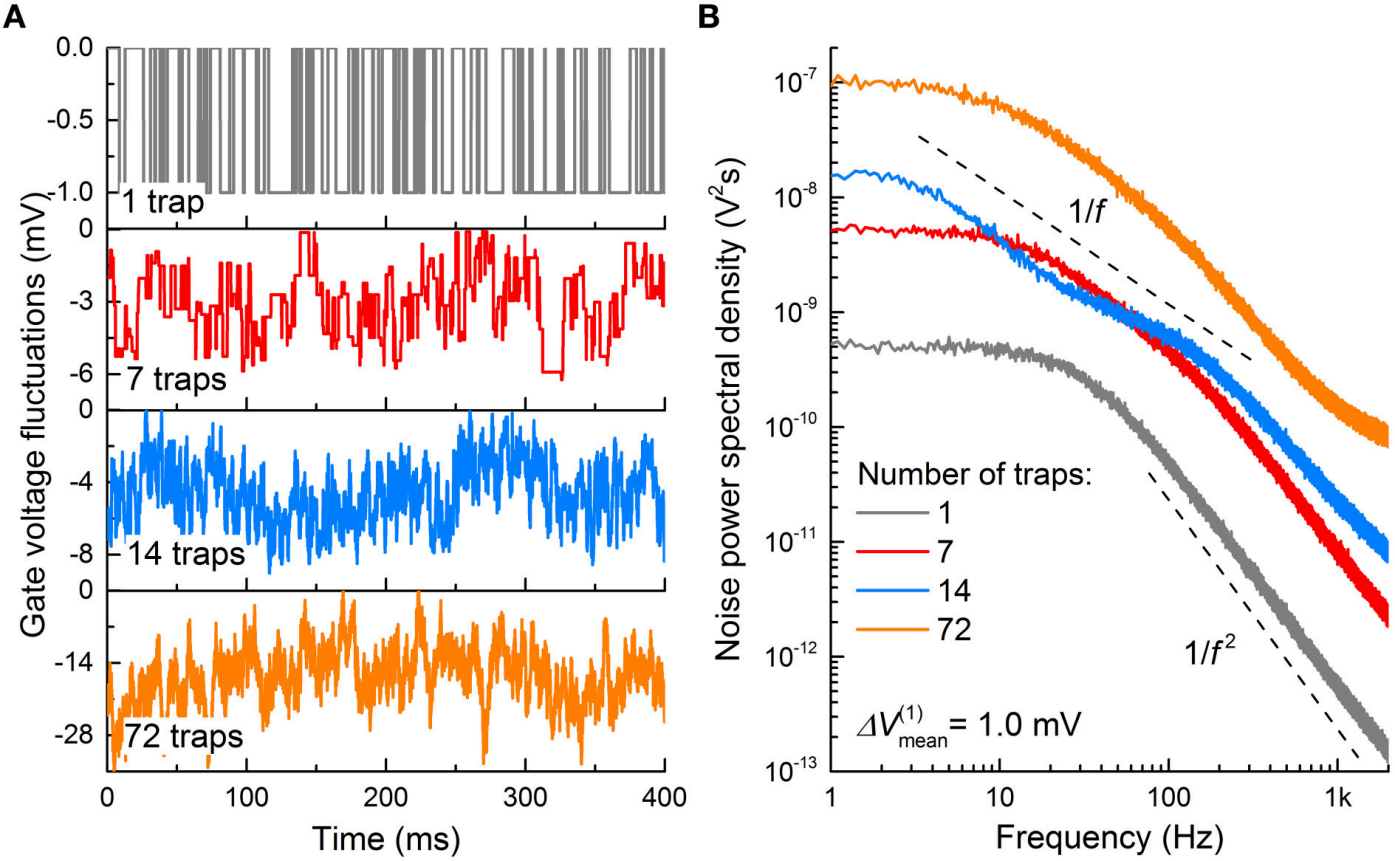
**Burst noise in MOSFET. (A)** Gate voltage fluctuations in an *n*MOS caused by electron trapping and detrapping events through interfacial charge traps. Average trapping and detrapping times for single-trap case (upper graph) were chosen to be 10 ms each, while in other cases they were taken from exponential distribution with a mean value of 10 ms. Δ*V*_fb_ owing to a single trap was also taken from exponential distribution with a mean value of 1.0 mV. **(B)** Noise PSD, evaluated for the temporal patterns in **(A)**.

The topmost panel in Figure 8A shows a typical fluctuation in *V*_g_ for *n*MOS (channel area: 120 × 60 nm^2^; gate oxide thickness: 2.5 nm) attributed to a single trap at the gate oxide/Si interface. Δ*V*_fb_ was 1.0 mV. Δ*V*_g_ is thus toggled between 0 and −1.0 mV upon the interaction between the trap and an electron; *V*_g_ is −1.0 mV when the trap is filled with an electron, and *V*_g_ is 0 otherwise. The corresponding PSD is plotted in Figure 8B, in which the PSD dispersion in the double logarithmic plot follows a power low with an exponent of −2 (Lorentzian PSD). The same holds for *p*MOSs with the same parameters, except that Δ*V*_g_ is toggled between 0 and 1.0 mV, and Δ*V*_g_ is 1.0 mV when the trap is filled with a hole.

The number of traps underneath the gate oxide is not necessarily one. Interfacial trap density *D*_it_ evaluates the average number of traps per unit area in the *n*-channel area; the larger *D*_it_, the more traps are likely present at the interface. The number of traps (7, 14, and 72 traps) markedly alters the fluctuation in *V*_g_, as shown in Figure 8A. An i.i.d. random Δ*V*_fb_ and *r*_t_ were assigned to each trap; both were drawn from exponential distribution functions with 〈Δ*V*_fb_〉 differing for different MOSFETs and a 〈τ_t_〉 of 10 ms, respectively. Additionally, each trap was assumed to interact with a single carrier. The method of noise generation is detailed in Materials and Methods. As shown, the *V*_g_ deviation from 0 increases with the number of traps because Δ*V*_fb_ proportionally increases with the number of filled traps. Notably, the PSD becomes close to flicker noise with the number of traps; for instance, 72 traps (orange PSD in Figure 8B) lead to a PSD approximately following an exponent of -1 in the double logarithmic plot. This is a consequence of the presence of multiple Lorentzian PSD functions (72 in total), i.e., superposition of these many PSD functions results in a 1/*f* PSD function (Campbell et al., 2009). Burst noise is thus a subset of flicker noise with regard to their origins. Long-channel MOSFETs often exhibit flicker noise attributed to the probable large number of traps at a given *D*_it_ (Uren et al., 1985). By contrast, deep-submicron MOSFETs likely include a few traps (or even a single trap); therefore, burst noise is predominantly observed (Hung et al., 1990).

**Figure 9:**
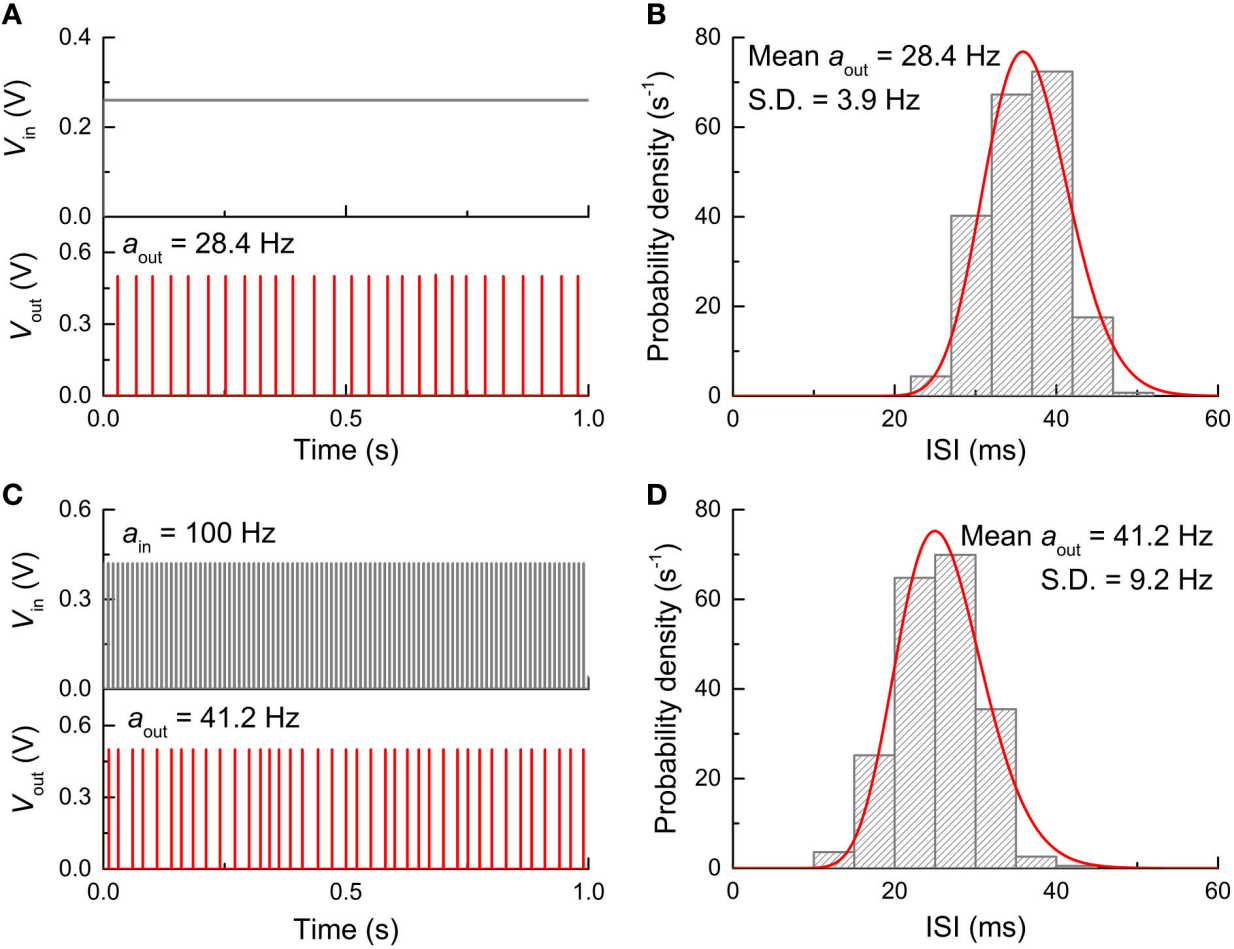
**Neuron firing in the presence of noise. (A)** Neuron firing when stimulated by a dc input voltage of 0.26 V. **(B)** ISI histogram for dc input and Gamma distribution function fitted to the ISI histogram. **(C)** Neuron firing when stimulated by a periodic spike train with a firing rate of 100 Hz and amplitude of 0.42 V. **(D)** ISI histogram for spiking input condition and Gamma distribution function fitted to the histogram.

**Figure 10:**
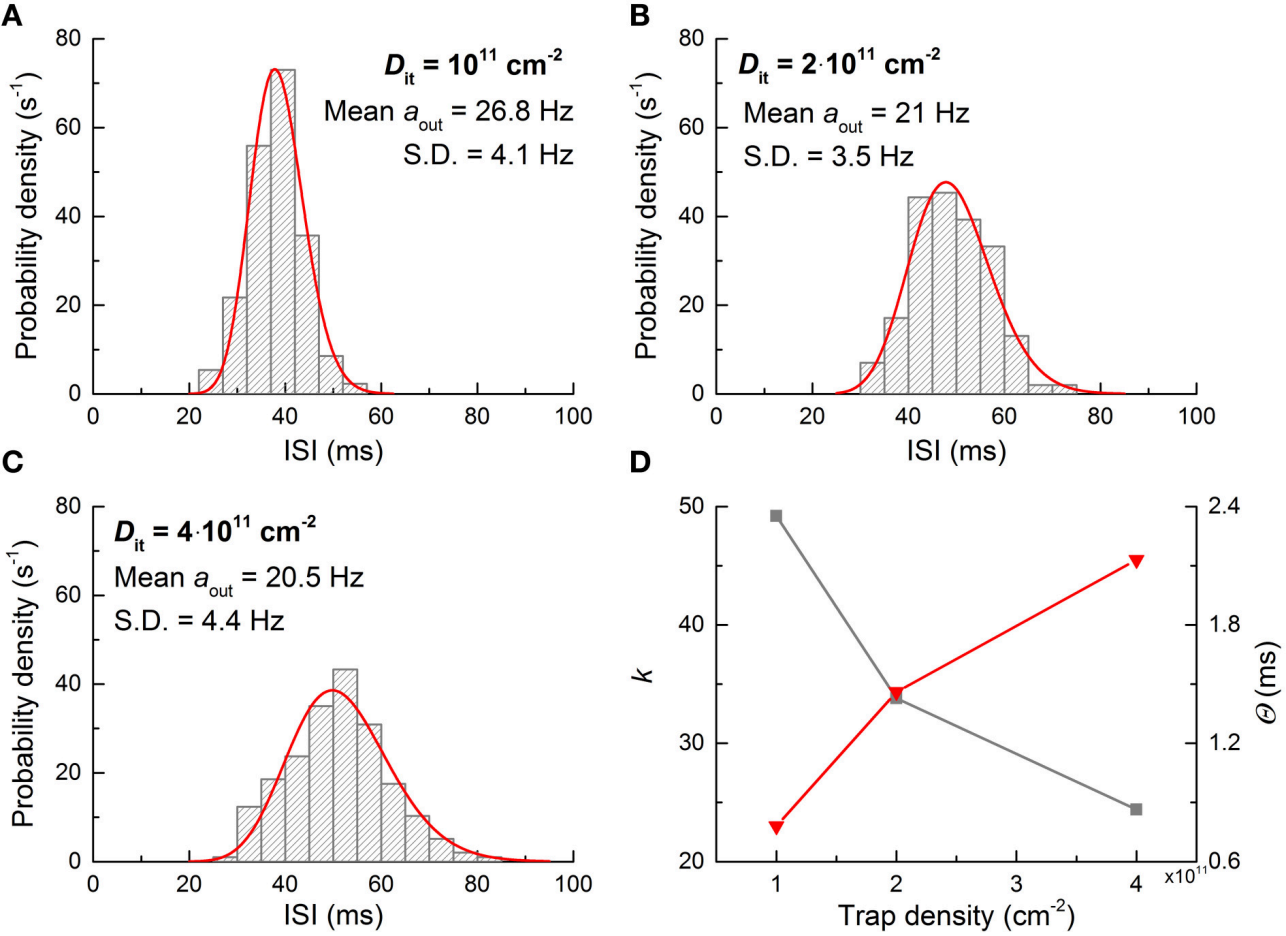
**ISI distribution with interfacial trap density. (A–C)** Neuron output ISI distributions for three different *D*_*it*_ values when it is subjected to a dc input of 0.26 V. **(D)** Gamma distribution parameters used for fitting histograms in **(A–C)**. The gray and red symbols denote *k* and Θ, respectively.

Eventually, the FGLIF neuron circuit was simulated taking into account the aforementioned types of noise. The circuit simulation was done for both dc input mode (*V*_in_: 0.26 V) and spike input mode (*a*_in_: 100 Hz, *V*_in_: 0.42 V) for direct comparison with the behaviors without noise shown in Figures 4, 5. Figure 9 displays the simulation results, including output spikes in time and ISI distribution. Note that *D*_it_ for all MOSFETs was set to 10^11^ cm^−2^ in the simulations. The ISI (τ) distribution apparently arises from the present noise as compared with the perfect periodicity of spikes (single ISI) for the noise-free neuron circuit. The ISI (τ) histogram for both input modes can be fitted well to a Gamma distribution function given by

(2) $$\begin{array}{cc} p\left(\tau \right)=\tau^{k-1}e^{-\tau /\theta }\;\cdot \;{\left(\theta^{k}\Gamma \left(k\right)\right)}^{-1}, & \;(2) \end{array}$$

where *k* and θ are fitting parameters that determine the shape and scale of the distribution, respectively (Maimon and Assad, 2009). As shown in Figure 8, the noise characteristic markedly varies upon the number of traps; therefore, the ISI distribution may change accordingly. To identify this effect, additional ISI histograms were obtained with different *D*_it_ values (10^11^, 2 × 10^11^, and 4 × 10^11^ cm^−2^), and the results are shown in Figure 10. The higher number of traps at the interface, the more widely the ISI is distributed. The decrease in the shape parameter *k* denotes an increase in spiking irregularity (Maimon and Assad, 2009), which is captured by the ISI widening. Note that in these simulations the contribution of each noise source to the overall noise effect cannot be distinguished mostly due to the physical origin of flicker and burst noise as mentioned earlier. Nevertheless, the power spectral density arising from interfacial charge traps becomes close to flicker noise with the number of charge traps (see Figure 8B), and thus the change in the ISI distribution with trap density in Figure 10 most likely reflects the distributional change upon the transition of a noise mechanism from burst-like to flicker-like noise. This transition widens a range of trapping and detrapping probabilities that are proportional to trapping and deptrapping rates as detailed in Section Materials and Methods. Consequently, a wide range of such rates is intertwined in the overall noise dynamics. Therefore, spiking regularity—parameterized by *k*—increases with trap density.

**Figure 11:**
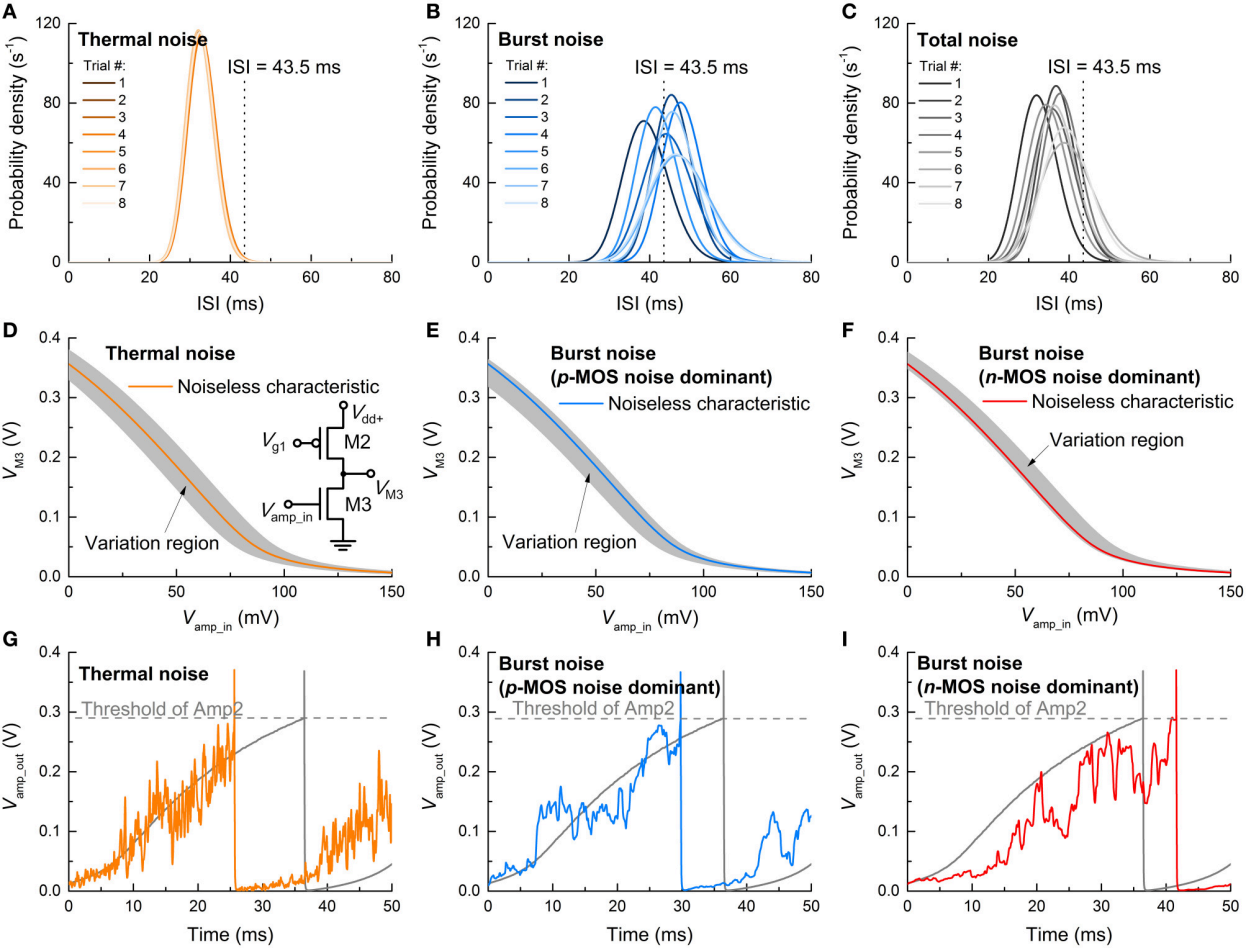
**ISI variation upon noise-type**. Output ISI distributions in the presence of **(A)** only thermal noise, **(B)** only burst noise, and **(C)** simultaneous thermal and burst noise at a constant dc input of 0.26 V. The dashed vertical lines at 43.5 ms indicate the ISI of noise-free spiking. The VTC of the input stage (M2–M3) of Amp1—shown in the inset—in the presence of **(D)** only thermal noise and only burst noise in **(E)** *p*MOS (M2) and **(F)** *n*MOS (M3). The shaded regions denote VTC variation ranges. The evolution of output of Amp1 (*V*_amp_out_) for cases of **(D–F)** is exemplified in **(G–I)**, respectively.

To identify the relative contributions of thermal and burst noise to the overall ISI distribution, further simulations were conducted by controlling the noise sources. Three cases were considered: thermal noise only, burst noise (subset of flicker noise) only, and simultaneous thermal and burst noise. Each case was examined from 10 independent simulations. The resulting ISI distributions (fitted to Gamma distribution functions) are shown in Figures 11A–C. As such, the thermal noise is determined by capacitance (see Equation 1) so that it is almost invariant through the trials. This is featured by the negligible trial-to-trial variation in the ISI distribution (Figure 11A). Notably, the average ISI shifts toward a lower value due to the thermal noise as seen in comparison with the noise-free case (ca. 43.5 ms) indicated by a dashed vertical line. By contrast, the burst noise markedly alters the ISI distribution upon trial (Figure 11B) because Δ*V*_fb_ and *r*_t_ values were sampled at random for each trial from exponential distributions (Yonezawa et al., 2013). The center of the distribution for each trial is scattered around the ISI of the noise-free case. In the present of simultaneous thermal and burst noise, the effects are superimposed (Figure 11C); the scattered positions of distributional centers relate to the burst noise, and their shift below 43.5 ms relates to the thermal noise. The noise-sensitive ISI distribution is mostly dictated by the noise in Amp1 (Figure 11B) whose input (*V*_amp_in_) directly evokes a spike. The thermal noise endows *V*_amp_in_ with a fluctuation around the noise-free *V*_amp_in_ so that the output from the input stage (M2–M3), i.e., *V*_M3_ in the inset of Figure 11D, fluctuates around the noise-free VTC (gray zone in Figure 11D). This fluctuation, in turn, affects the input into the following stage (M4–M5) in conjunction with its own noise. A typical reduction in ISI due to thermal noise is shown in Figure 11G. The output overshoot accelerates spiking (orange line) that precedes the noise-free spiking (gray line) by ~10 ms. Therefore, the thermal noise generally shorten the ISI.

The effect of burst noise on Amp1 differs for *p*MOS and *n*MOS. For *p*MOS, the trapped holes at the gate oxide/Si interface leads to a negative shift in *V*_fb_, i.e., Δ*V*_fb_ < 0; therefore, when the burst noise in the *p*MOS is solely present in the input stage, the VTC shifts as a whole as shown in Figure 11E. Such a shift causes a reduction in Vamp_in to output the same *V*_M3_ as for the noise-free case so that the ISI is reduced—similar to the thermal noise effect. An example is shown in Figure 11H. By contrast, for *n*MOS, the electrons trapped at the channel interface elevate *V*_fb_, i.e., Δ*V*_fb_ > 0, indicating a right shift in the VTC as a whole (Figure 11F). Consequently, Amp1 needs higher *V*_amp_in_ than the noise-free case to output the same *V*_M3_ as for the noise-free case, implying larger ISI. An example of this case is plotted in Figure 11I.

## Discussion

**Figure 12:**
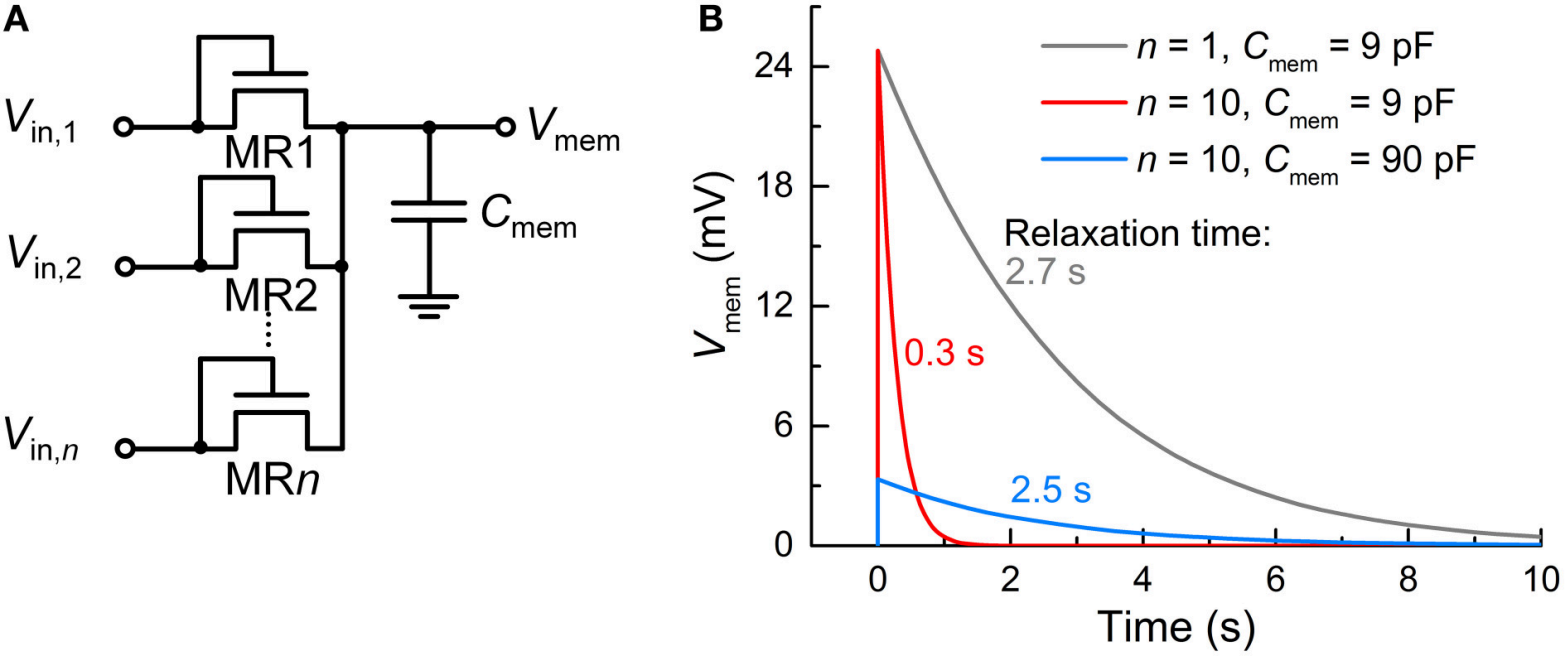
**Comparison with switched-capacitor integrator. (A)** Switched-capacitor integrator circuit with *n* input MOSFET terminals (MR1–MR*n*). **(B)** Relaxation of *V*_mem_—parameterizing integration—with time for different *n*'s (1 and 10) and capacitance values of C_mem_ (9 and 90 pF). An input spike (*V*_in_ = 0.5 V, *t*_isp_ = 10 μs) was applied to a single tunnel junction at 0 s and the others were grounded.

Recall that unlike capacitor-based integrators, the FG integrator in the proposed FGLIF neuron has the characteristic relaxation time defined by quantum mechanical tunneling dynamics through the tunnel barrier. The different basis of charge integration largely mitigates a severe need for high capacitance in favor of a biologically plausible timescale. In order to highlight the scalability of the proposed FGLIF neuron circuit (particularly the FG integrator), the FG integrator was compared with a switched-capacitor integrator comprising *n* MOSFET switches (MR1–MR*n*) and one capacitor *C*_mem_. The switched-capacitor integrator is illustrated in Figure 12A. Note that the MOSFETs have the gate and drain shorted in order to realize “fast charging and slow discharging.” They are switched on upon the application of a voltage pulse to the shorted terminal.

All MOSFETs in this integrator were of the same size (channel length/width: 60/120 nm) as the tunnel junctions and FG transistor M1 in the FG integrator. The circuit simulation results indicate a need for remarkably high capacitance—more than three orders of magnitude higher than that of C_FG_ (6 fF)—to achieve a relaxation time of a few seconds for the FG integrator (compare Figure 12B with Figure 2B). For a fair comparison, the input voltage pulse was identical to that of the FG integrator shown in Figure 2 (amplitude: 0.5 V, width: 10 μs). Furthermore, the use of multiple terminals (*n* = 10) significantly reduces the relaxation time by approximately one order of magnitude in light of a decrease in the equivalent resistance owing to the MOSFETs being in parallel (Figure 12B). The same holds for the FG integrator as addressed in Section Circuit Simulations, and a relaxation time of a few seconds was recovered by introducing a slightly thicker tunnel barrier. By contrast, the switched-capacitor integrator requires a higher capacitance to avoid such a large decrease in the relaxation time, e.g., 90 pF to endow the integrator with a relaxation time of ~2.5 s, as shown in Figure 12B. However, the high capacitance value retards not only the discharging (relaxation) but also the charging; therefore, the *V*_mem_ maximum (ca. 24.8 mV) was not reached during the period of a single spike.

Such high capacitance in this simple integrator is hardly affordable in integrated circuits, delimiting the scalability. It is revealed that realizing a capacitance of a few pF requires a few hundred μm^2^, which cannot fit in with the framework for scaling down. As addressed in Section Circuit Simulations, *C*_FG_ of 6 fF in capacitance was placed on M1 (channel length/width: 60/120 nm), and thus the FG integrator scheme offers great scalability, which is a definite advantage over switched-capacitor integrators. Of course, several strategies for reducing the capacitor area are likely available, e.g., introducing high-*k* dielectrics, or HfO_2_ and/or three-dimensional capacitors (Kim et al., 2010). However, such attempts may cause additional complexity in chip fabrication and a consequent fabrication cost that may outweigh the benefits. Alternatively, maximizing the resistance of the switch in a switched-capacitor integrator equally enables a long relaxation time, as demonstrated by Noack et al. (2015). Another strategy based on capacitor-based integration by Qiao et al. (2015) accomplished a time constant of a few tens of milliseconds using a 1 pF capacitor. Nevertheless, our FG-based strategy may be a potential alternative to the capacitor-based integration for further scaling down neurons.

One of the main concerns for flash memories is reliability, particularly in the endurance of the FG transistor. Poor endurance—although sufficient for flash memory applications with a write endurance of 10^4^–10^5^ times—is mainly ascribed to a high write voltage in exchange for better data retention (Cappelletti and Modelli, 1999). Given the circumstances for the operation of the FG integrator (receiving a train and/or burst of a number of spikes), the poor endurance is perhaps a significant obstacle. Low neuronal activity (as low as biological neurons) is preferred partly on these grounds. Fortunately, unlike with flash memory, we deliberately allow data (charge on the FG) loss for a relaxation time of a few seconds, so that the requisite write and erase voltages are much lower, i.e., ±0.5 V in the proposed circuit. This low voltage is mostly applied to the tunnel junction; the voltage across *C*_FG_ is merely 47 mV (maximum *V*_FG_ shown in Figure 4B) at most, and the gate oxide is subject to even less voltage with regard to the additional voltage drop in the Si channel underneath. The simulation results show ~90 electrons that tunnel through the gate oxide in a single spiking period, i.e., one output spike (amplitude: 0.5 V, width: 25 μs) and one ISI, which is equivalent to a charge density of ~2 × 10^−7^ C/cm^2^. Thus, such low voltage likely alleviates the burden on the FG, rendering the FG fairly endurable (Wann and Hu, 1995).

Significant improvement in the reliability of flash memory occurred by replacing the standard silicon FG with an insulating SiN_*x*_ one, forming so-called silicon-oxide-nitride-oxide-silicon (SONOS; Chan et al., 1987). The insulating SiN_*x*_ tolerates shorting paths embedded in the tunnel barriers to a greater degree than the standard SOSOS (silicon instead of SiN_*x*_ in SONOS; Chan et al., 1987). However, provided that the synapse transistor stretches the FG to the separate tunnel junctions, the conductance of the FG is of significant concern. To put it precisely, the highly resistive SiN_*x*_ FG barely alters *V*_FG_ in M1 upon a charge transfer through the separate tunnel junctions. The standard SOSOS is therefore suitable for the FG integrator. The aforementioned low write and erase voltages that suffice for the FG integrator likely support the reliability of SOSOS.

As such, the number of the interfacial charge traps is most likely proportional to the channel size, and the effect of a single trap on Δ*V*_fb_ is predicted to become more significant as the channel size shrinks (Fukuda et al., 2007; Miki et al., 2012). The continuum-based model in this study may not precisely elucidate the effect of interfacial traps on Δ*V*_fb_ in a state-of-the-art nanoscale MOSFET. However, discrete models that are more suitable for dealing with a few traps also indicate the same tendency as for the continuum model (Fukuda et al., 2007; Miki et al., 2012). Experimental studies have pointed to burst noise as a dominant type of noise in nanoscale MOSFETs (Tega et al., 2009; Miki et al., 2012).

Noise in integrated circuits is inevitable as such, and the noise induces irregular spiking patterns (Figures 9, 10). The ISI histograms are nicely fitted to Gamma distribution functions with different fitting parameters akin to biological neurons, e.g., parietal neurons (Bair et al., 1994; Maimon and Assad, 2009). The integrated FGLIF neuron circuit in practice may differ in noise characteristics from our theoretical one. Even among integrated FGLIF neuron circuits, the difference in noise is most likely evident because the variables that relate to noise, e.g., *D*_it_, Δ*V*_fb_, and *r*_t_, are markedly dependent on the details of fabrication methods. It is obvious that traps are incorporated in a MOSFET to some extent, and they endow the MOSFET with noise. A fundamental question arises as to whether such irregular spiking helps neural processing or is an obstacle to neural processing. Within the framework of stochastic electronics (Chen et al., 2010; Hamilton et al., 2014), such irregular spiking is necessary, and imperfections such as charge traps are required for the disorder. For instance, it has turned out that the noise given to a VLSI Hodgkin-Huxley neuron enhances signal modulation (Chen et al., 2010). Nevertheless, answering this fundamental question is beyond the scope of this paper, so we leave the question open.

## Conclusion

We proposed an FGLIF neuron circuit, which likely achieves input-dependent output spiking activity within a biologically plausible range with a capacitor of merely 6 fF. Such low capacitance offers a great opportunity for scaling down the FGLIF neuron circuit while maintaining the activity scale. In addition, given the subthreshold operation of most MOSFETs in the circuit, spiking consumes less than 30 pW of power irrespective of spiking activity, rendering the FGLIF neuron very suitable for large-scale SNNs. In addition, the FGLIF neuron circuit is fully compatible with standard CMOS technology, which is of great benefit. Unavoidable noise in the circuit leads to distributional features of ISIs. The ISI distribution was fitted to a Gamma distribution function that has often described the ISI distribution of neocortical neurons.

## Author contributions

VK conceived the idea and performed the circuit calculations together with HL, JS, and BC, GK, SK, and IK also conceived the idea and support the calculations. DJ initiated and supervised this study and wrote the manuscript. All authors discussed the results and contributed to the refinement of the manuscript.

### Conflict of interest statement

The authors declare that the research was conducted in the absence of any commercial or financial relationships that could be construed as a potential conflict of interest.

## References

[B1] Arthur J. V. Boahen K. (2004). Recurrently connected silicon neurons with active dendrites for one-shot learning, in IEEE International Joint Conference on Neural Networks, Vol. 3 (Budapest), 1699–1704.

[B2] Arthur J. V. Boahen K. (2007). Synchrony in silicon: the gamma rhythm. IEEE Trans. Neural Netw. 18, 1815–1825. 10.1109/TNN.2007.900238 18051195

[B3] Azghadi M. R. Iannella N. Al-Sarawi S. F. Indiveri G. Abbott D. (2014). Spike-based synaptic plasticity in silicon: design, implementation, application, and challenges. Proc. IEEE 102, 717–737. 10.1109/JPROC.2014.2314454

[B4] Bair W. Koch C. Newsome W. Britten K. (1994). Power spectrum analysis of bursting cells in area MT in the behaving monkey. J. Neurosci. 14, 2870–2892. 8182445 10.1523/JNEUROSCI.14-05-02870.1994 PMC6577471

[B5] Bartolozzi C. Indiveri G. (2007). Synaptic dynamics in analog VLSI. Neural Comput. 19, 2581–2603. 10.1162/neco.2007.19.10.2581 17716003

[B6] Brink S. Nease S. Hasler P. (2013). Computing with networks of spiking neurons on a biophysically motivated floating-gate based neuromorphic integrated circuit. Neural Netw. 45, 39–49. 10.1016/j.neunet.2013.02.011 23541925

[B7] Burkitt A. N. (2006). A review of the integrate-and-fire neuron model: I. Homogeneous synaptic input. Biol. Cybern. 95, 1–19. 10.1007/s00422-006-0068-6 16622699

[B8] Campbell J. P. Qin J. Cheung K. P. Yu L. C. Suehle J. S. Oates A. (2009). Random telegraph noise in highly scaled nMOSFETs, in Reliability Physics Symposium, 2009 IEEE International (Montreal, QC), 382–388.

[B9] Cao K. M. Lee W. C. Liu W. Jin X. Su P. Fung S. K. H. (2000). BSIM4 gate leakage model including source-drain partition, in Electron Devices Meeting, 2000. IEDM′00. Technical Digest. International (San Francisco, CA), 815–818.

[B10] Cappelletti P. Modelli A. (1999). Flash Memory Reliability. New York, NY: Springer.

[B11] Chan T. Y. Young K. K. Hu C. (1987). A true single-transistor oxide-nitride-oxide EEPROM device. Electron Device Lett. 8, 93–95.

[B12] Chen H. Saïghi S. Buhry L. Renaud S. (2010). Real-time simulation of biologically realistic stochastic neurons in VLSI. IEEE Trans. Neural Netw. 21, 1511–1517. 10.1109/TNN.2010.2049028 20570768

[B13] Chong Z. Y. Sansen W. (2013). Low-Noise Wide-Band Amplifiers in Bipolar and CMOS Technologies, Vol. 117. New York, NY: Springer Science & Business Media.

[B14] Dayan P. Abbott L. F. (2001). Theoretical Neuroscience, Vol. 806 Cambridge, MA: MIT Press.

[B15] Diorio C. Hasler P. Minch B. A. Mead C. (1998). Floating-gate MOS synapse transistors, in Neuromorphic Systems Engineering, ed Lande T. S. (Norwell, MA: Kluwer Academic Publishers), 315–337.

[B16] Dunga M. V. Xi X. He J. Liu W. Cao K. M. Jin X. (2006). BSIM 4.6.0 MOSFET Model. Berkeley, CA: University of California.

[B17] Edwards R. T. Cauwenberghs G. (2000). Synthesis of log-domain filters from first-order building blocks, in Research Perspectives on Dynamic Translinear and Log-Domain Circuits, eds Serdijn W. A. Mulder J. (New York, NY: Springer), 71–80.

[B18] Eliasmith C. Anderson C. H. (2004). Neural Engineering: Computation, Representation, and Dynamics in Neurobiological Systems. Cambridge, MA: MIT press.

[B19] Eliasmith C. Stewart T. C. Choo X. Bekolay T. DeWolf T. Tang Y.. (2012). A large-scale model of the functioning brain. Science 338, 1202–1205. 10.1126/science.1225266 23197532

[B20] Fukuda K. Shimizu Y. Amemiya K. Kamoshida M. Hu C. (2007). Random telegraph noise in flash memories-model and technology scaling, in Electron Devices Meeting, 2007. IEDM 2007. IEEE International (Washington, DC), 169–172.

[B21] Gerstner W. Kistler W. M. (2002). Spiking Neuron Models: Single Neurons, Populations, Plasticity. Cambridge: Cambridge University Press.

[B22] Gordon C. Farquhar E. Hasler P. (2004). A family of floating-gate adapting synapses based upon transistor channel models, in Proceedings of the 2004 International Symposium on Circuits and Systems, 2004. ISCAS′ 04, Vol. 1 (Vancouver, BC), I-317–I-320.

[B23] Hamilton T. J. Afshar S. van Schaik A. Tapson J. (2014). Stochastic electronics: a neuro-inspired design paradigm for integrated circuits. Proc. IEEE 102, 843–859. 10.1109/JPROC.2014.2310713

[B24] Hasler P. Diorio C. Minch B. A. Mead C. (1994). Single transistor learning synapses, in Advances in Neural Information Processing Systems 7, eds Tesauro G. Touretzky D. S. (Cambridge, MA: MIT Press), 817–824.

[B25] Hodgkin A. L. Huxley A. F. (1952). A quantitative description of membrane current and its application to conduction and excitation in nerve. J. Physiol. 117, 500–544. 12991237 10.1113/jphysiol.1952.sp004764 PMC1392413

[B26] Hung K. K. Chenming H. Cheng Y. C. (1990). Random telegraph noise of deep-submicrometer MOSFET's. IEEE Electron Device Lett. 11, 90–92.

[B27] Indiveri G. Chicca E. Douglas R. (2006). A VLSI array of low-power spiking neurons and bistable synapses with spike-timing dependent plasticity. IEEE Trans. Neural Netw. 17, 211–221. 10.1109/TNN.2005.860850 16526488

[B28] Indiveri G. Linares-Barranco B. Hamilton T. J. Van Schaik A. Etienne-Cummings R. Delbruck T.. (2011). Neuromorphic silicon neuron circuits. Front. Neurosci. 5:73. 10.3389/fnins.2011.00073 21747754 PMC3130465

[B29] Izhikevich E. M. (2003). Simple model of spiking neurons. IEEE Trans. Neural Netw. 14, 1569–1572. 10.1109/TNN.2003.820440 18244602

[B30] Jeong D. S. Hwang C. S. (2005). Tunneling current from a metal electrode to many traps in an insulator. Phys. Rev. B71:165327 10.1103/PhysRevB.71.165327

[B31] Jeong D. S. Kim I. Ziegler M. Kohlstedt H. (2013). Towards artificial neurons and synapses: a materials point of view. RSC Adv. 3, 3169–3183. 10.1039/C2RA22507G

[B32] Jeong D. S. Thomas R. Katiyar R. S. Scott J. F. Kohlstedt H. Petraru A.. (2012). Emerging memories: resistive switching mechanisms and current status. Rep. Prog. Phys. 75:076502. 10.1088/0034-4885/75/7/076502 22790779

[B33] Kim S. K. Lee S. W. Han J. H. Lee B. Han S. Hwang C. S. (2010). Capacitors with an equivalent oxide thickness of <0.5 nm for nanoscale electronic semiconductor memory. Adv. Funct. Mater. 20, 2989–3003. 10.1002/adfm.201000599

[B34] Lee W. C. Hu C. (2001). Modeling CMOS tunneling currents through ultrathin gate oxide due to conduction-and valence-band electron and hole tunneling. Electron Devices IEEE Trans. 48, 1366–1373. 10.1109/16.930653

[B35] Lee Y. J. Lee J. Kim Y. B. Ayers J. Volkovskii A. Selverston A. (2004). Low power real time electronic neuron VLSI design using subthreshold technique, in Proceedings of the 2004 International Symposium on Circuits and Systems, 2004. ISCAS′04, Vol. 4 (Vancouver, BC), IV-744–IV-747.

[B36] Lim H. Kornijcuk V. Seok J. Y. Kim S. K. Kim I. Hwang C. S.. (2015). Reliability of neuronal information conveyed by unreliable neuristor-based leaky integrate-and-fire neurons: a model study. Sci. Rep. 5, 9776. 10.1038/srep09776 25966658 PMC4429369

[B37] Mahowald M. Douglas R. (1991). A silicon neuron. Nature 354, 515–518. 1661852 10.1038/354515a0

[B38] Maimon G. Assad J. A. (2009). Beyond poisson: increased spike-time regularity across primate parietal cortex. Neuron 62, 426–440. 10.1016/j.neuron.2009.03.021 19447097 PMC2743683

[B39] Markram H. (2006). The blue brain project. Nat. Rev. Neurosci. 7, 153–160. 10.1038/nrn1848 16429124

[B40] Mead C. (1989). Analog VLSI and Neural Systems. Reading, MA: Addison-Wesley.

[B41] Mead C. (1990). Neuromorphic electronic systems. Proc. IEEE 78, 1629–1636.

[B42] Merolla P. A. Arthur J. V. Alvarez-Icaza R. Cassidy A. S. Sawada J. Akopyan F.. (2014). A million spiking-neuron integrated circuit with a scalable communication network and interface. Science 345, 668–673. 10.1126/science.1254642 25104385

[B43] Miki H. Tega N. Frank D. J. Bansal A. Kobayashi M. Cheng K. (2012). Statistical measurement of random telegraph noise and its impact in scaled-down high-k/metal-gate MOSFETs, in Electron Devices Meeting, 2012. IEDM 2012. IEEE International (San Francisco, CA), 450–453.

[B44] Noack M. Partzsch J. Mayr C. G. Hänzsche S. Scholze S. Höppner S.. (2015). Switched-capacitor realization of presynaptic short-term-plasticity and stop-learning synapses in 28 nm CMOS. Front. Neurosci. 9:10. 10.3389/fnins.2015.00010 25698914 PMC4313588

[B45] Nyquist H. (1928). Thermal agitation of electric charge in conductors. Phys. Rev. 32, 110–113.

[B46] Polsky A. Mel B. W. Schiller J. (2004). Computational subunits in thin dendrites of pyramidal cells. Nat. Neurosci. 7, 621–627. 10.1038/nn1253 15156147

[B47] Qiao N. Mostafa H. Corradi F. Osswald M. Stefanini F. Sumislawska D.. (2015). A reconfigurable on-line learning spiking neuromorphic processor comprising 256 neurons and 128K synapses. Front. Neurosci. 9:141. 10.3389/fnins.2015.00141 25972778 PMC4413675

[B48] Rahimi K. Diorio C. Hernandez C. Brockhausen M. D. (2002). A simulation model for floating-gate MOS synapse transistors, in IEEE International Symposium on Circuits and Systems, 2002. ISCAS 2002, Vol. 2 (Phoenix-Scottsdale, AZ), II-532–II-535.

[B49] Ramakrishnan S. Wunderlich R. Hasler J. George S. (2013). Neuron array with plastic synapses and programmable dendrites. IEEE Trans. Biomed. Circuits Syst. 7, 631–642. 10.1109/BioCAS.2012.6418412 24144669

[B50] Ranuárez J. C. Deen M. J. Chen C. H. (2006). A review of gate tunneling current in MOS devices. Microelectronics Reliab. 46, 1939–1956. 10.1016/j.microrel.2005.12.006

[B51] Riggert C. Ziegler M. Schroeder D. Krautschneider W. H. Kohlstedt H. (2014). MemFlash device: floating gate transistors as memristive devices for neuromorphic computing. Semiconductor Sci. Tech. 29, 104011–104019. 10.1088/0268-1242/29/10/104011

[B52] Sarpeshkar R. Delbruck T. Mead C. (1993). White noise in MOS transistors and resistors. Circuits Devices Mag. IEEE 9, 23–29. 10.1109/101.261888

[B53] Soni R. Petraru A. Meuffels P. Vavra O. Ziegler M. Kim S. K.. (2014). Giant electrode effect on tunnelling electroresistance in ferroelectric tunnel junctions. Nat. Commun. 5, 5414. 10.1038/ncomms6414 25399545

[B54] Tega N. Miki H. Ren Z. D'Emic C. P. Zhu Y. Frank D. J. (2009). Reduction of random telegraph noise in high-κ/metal-gate stacks for 22 nm generation FETs, in Electron Devices Meeting, 2009. IEDM 2009. IEEE International (Baltimore, MD), 771–774.

[B55] Tenore F. Vogelstein R. J. Etienne-Cummings R. Cauwenberghs G. Hasler P. (2006). A floating-gate programmable array of silicon neurons for central pattern generating networks, in 2006 IEEE International Symposium on Circuits and Systems, 2006. ISCAS 2006. Proceedings (Island of Kos), 3157–3160.

[B56] Uren M. J. Day D. J. Kirton M. (1985). 1/*f* and random telegraph noise in silicon metal−oxide−semiconductor field−effect transistors. Appl. Phys. Lett. 47, 1195–1197. 10.1103/physrevb.37.8346 9944172

[B57] Van Schaik A. Jin C. T. McEwan A. L. Hamilton T. J. (2010). A log-domain implementation of the Izhikevich neuron model, in Proceedings of 2010 IEEE International Symposium on Circuits and Systems (ISCAS) (Paris), 4253–4256.

[B58] Wann C. H. Hu C. (1995). High endurance ultra-thin tunnel oxide for dynamic memory application, in Electron Devices Meeting, 1995. IEDM′95., International (IEEE) (Washington, DC), 867–870.

[B59] Wijekoon J. H. Dudek P. (2008). Compact silicon neuron circuit with spiking and bursting behaviour. Neural Netw. 21, 524–534. 10.1016/j.neunet.2007.12.037 18262751

[B60] Yonezawa A. Teramoto A. Obara T. Kuroda R. Sugawa S. Ohmi T. (2013). The study of time constant analysis in random telegraph noise at the subthreshold voltage region, in 2013 IEEE International Reliability Physics Symposium (Anaheim, CA), XT.11.1–XT.11.6. 10.1109/IRPS.2013.6532126

